# iPS-Derived Early Oligodendrocyte Progenitor Cells from SPMS Patients Reveal Deficient In Vitro Cell Migration Stimulation

**DOI:** 10.3390/cells9081803

**Published:** 2020-07-29

**Authors:** Lidia Lopez-Caraballo, Jordi Martorell-Marugan, Pedro Carmona-Sáez, Elena Gonzalez-Munoz

**Affiliations:** 1 Laboratory of Cell Reprogramming (LARCEL), Andalusian Centre for Nanomedicine and Biotechnology-BIONAND, 29590 Málaga, Spain; llc.cellreprogramming@gmail.com; 2 Bioinformatics Unit. GENYO, Centre for Genomics and Oncological Research: Pfizer/University of Granada/Andalusian Regional Government, PTS Granada, E-18016 Granada, Spain; jordi.martorell@genyo.es (J.M.-M.); pedro.carmona@genyo.es (P.C.-S.); 3 Atrys Health, 08025 Barcelona, Spain; 4 Department of Statistics. University of Granada, 18071 Granada, Spain; 5 Department of Cell Biology, Genetics and Physiology, University of Málaga, 29071 Málaga, Spain; 6 Networking Research Center on Bioengineering, Biomaterials and Nanomedicine, (CIBER-BBN), 29071 Málaga, Spain

**Keywords:** secondary progressive multiple sclerosis (SPMS), disease modeling, iPSCs, oligodendrocyte progenitor cells (OPCs), secretome, cell migration

## Abstract

The most challenging aspect of secondary progressive multiple sclerosis (SPMS) is the lack of efficient regenerative response for remyelination, which is carried out by the endogenous population of adult oligoprogenitor cells (OPCs) after proper activation. OPCs must proliferate and migrate to the lesion and then differentiate into mature oligodendrocytes. To investigate the OPC cellular component in SPMS, we developed induced pluripotent stem cells (iPSCs) from SPMS-affected donors and age-matched controls (CT). We confirmed their efficient and similar OPC differentiation capacity, although we reported SPMS-OPCs were transcriptionally distinguishable from their CT counterparts. Analysis of OPC-generated conditioned media (CM) also evinced differences in protein secretion. We further confirmed SPMS-OPC CM presented a deficient capacity to stimulate OPC in vitro migration that can be compensated by exogenous addition of specific components. Our results provide an SPMS-OPC cellular model and encouraging venues to study potential cell communication deficiencies in the progressive form of multiple sclerosis (MS) for future treatment strategies.

## 1. Introduction

Multiple sclerosis (MS) is a chronic central nervous system (CNS) inflammatory demyelinating disease and the leading cause of non-traumatic acquired disability in young adults.

Although multiple sclerosis has been classified as a nonhereditary disease in the past, converging evidence supports the importance of genetic determinants for MS etiology. The disease is influenced by the genetic constitution of the individual, and it has been shown that there are genes that are associated with an increased risk of contracting the disease [1,2,3], although the functional implications of these associated variants are mostly unknown, suggesting a substantial potential for further discovery.

Although the origin of MS is idiopathic, it has traditionally been classified as an autoimmune inflammatory disease of the CNS white matter. Most treatments to date have focused on the inflammatory component of the disease, with a partial effect on relapse rates [4] but very low impact on the progressive phase of the disease, which is mostly associated with demyelination and axonal damage independent of inflammation [5,6].

Even if in the majority of relapsing-remitting clinical phase (RRMS) patients in early stages of the disease, remyelination and functional recovery occurs, this initial relapsing-remitting course evolves years later into a secondary progressive MS (SPMS) of uninterrupted disease progression, with limited remyelination that fails due to still largely unknown reasons, leading to a worsening of symptoms as a result of axonal injury and neuronal loss [7,8]. A proportion of MS patients (up to 15%) enter directly into the progressive phase without experiencing initial relapses, known as primary progressive MS (PPMS) [5]. Significantly, the mean age of onset of SPMS and PPMS is similar, approximately 40 years, and very little is known about the etiology of such disease progression with chronic CNS demyelination and neurodegeneration with minimal remyelination [9,10,11]. Since oligodendrocyte precursor cells (OPCs) are detected in MS lesions, it would suggest that the endogenous repair mechanisms that normally respond to damaged myelin are defective in the course of the disease.

Most of the data contributing to the understanding of the cellular and molecular progressive pathogenesis of MS research have come from in vivo mouse models either directly or through tissue cultivation in vitro [12]. However, the findings in murine models have been difficult to validate in MS patients, primarily because the majority of studies of demyelinating lesions in humans are generally limited to tissue postmortem patients [7,13]. The generation of human cellular models of specific cell types with induced pluripotent stem cell (iPSC) technology has opened a promising field in disease modeling and can greatly contribute to understanding the progressive phase of the disease [14,15,16].

Identification of key factors involved in successful migration, proliferation, differentiation, and myelination of OPCs may reveal new strategies for the treatment of the progressive form of the disease.

In this study, we report successful generation of SPMS-iPSCs from affected (and control, nonaffected) donors and further efficient OPC-like cell differentiation, although proliferating early OPCs show a specific and different expression profile compared to control cells. We conducted a proteomic analysis to provide information on misregulated proteins present in the SPMS-OPC secretome and showed functional evidence of its deficient effect on stimulation of OPC in vitro migration. Our data suggest SPMS may have a relevant component related to defective cell communication for early OPC migration.

## 2. Materials and Methods

### 2.1. Derivation of Human Adult Somatic Samples

All samples were collected and processed after obtaining donors’ informed consent via the Spanish Regional Biobank “*Biobanco del Sistema Publico Andaluz*” (BSSPA) and with clearances from the Stem Cell Ethical Committee and Review Board of the National Research Ethics Service (PR-02-2018). SPMS donors were subjected to regular interferon beta-1b treatment with no further pharmacological treatment at the time of sample acquisition (Table 1).

All cultures were maintained at 37 °C in a humidified atmosphere containing 5% CO_2_. All experiments were performed in passage 2–4. The specific protocol isolation used is described below:

Menstrual blood-derived stromal cells (MnSCs) were obtained from menstrual blood of SPMS and healthy volunteers at the peak of flow. Cells were centrifuged and submitted to the Ficoll (Histopaque 1077-Sigma, St. Louis, MO, USA) gradient following the manufacturer’s instructions. Mononuclear cells were culture in DMEM-F12 containing 10% FBS, 1 × NEAA, 1 × L-Glutamine, penicillin, and streptomycin (Thermo Scientific, Waltham, MA, USA) [17].

All cell lines were regularly tested for mycoplasma using PCR validation (Venor GeM Classic, Minerva Biolabs, Berlin, Germany) and found to be negative.

### 2.2. Vectors

DNA vectors pMX-GFP, pMX-OCT4, pMX-SOX2, pMX-KLF4, and pMX-cMYC (h.sapiens) were purchased from Addgene (Watertown, MA, USA).

### 2.3. Cell Culture of iPSCs

Generated iPS cells were cultured in standard human embryonic stem (hES) cell culture media (DMEM/F12 containing 20% KSR, 10ng/mL of human recombinant basic fibroblast growth factor (bFGF), 1 × NEAA, 1 × L-Glutamine, 5.5 mM 2-ME, penicillin and streptomycin (all from Thermo Scientific, Waltham, MA, USA)) on top of mitomycin-C (Sigma, St. Louis, MO, USA) treated mouse fibroblasts and picked mechanically, as previously described [18]. All cell lines were regularly tested for mycoplasma using PCR validation (Venor GeM Classic, Minerva Biolabs, Berlin, Germany) and found to be negative.

### 2.4. Production of Viral Supernatants

For retro vectors, Hek293T cells were plated at 90% cell confluence in a 10-cm dish. The next day, cells were transfected with 10 μg viral vector, 7 μg Gag-Pol vector (Addgene, Watertown, MA, USA), and 3 μg VSV-G plasmid (Addgene, Watertown, MA, USA) using the polyethyleneimine method. The supernatant was collected 24 h and 48 h post-transfection and filtered through 45-mm pore size filters. Tittering was performed on Hek293Ts. 5 mL of unconcentrated viral supernatant was used to transduce 25,000 cells in the presence of 4 μg/mL polybrene (Sigma, St. Louis, MO, USA).

### 2.5. Reprogramming Assays

Low passage (passage 2–4) MnSCs were seeded at 100,000 cells/well and transduced with retroviral supernatants encoding OSK factors (pMX-OCT4, pMX-Sox2, pMX-KLF4) in the presence of 4 μg/mL polybrene (Sigma, St. Louis, MO, USA). Twenty-four hours later, cells were replated onto six-well plates on a feeder layer of mitomycin C-treated mouse embryonic fibroblasts (Millipore, Burlington, MA, USA). The medium was changed to hES medium daily. Colonies appeared at day 18–28 after transduction. TRA-160+ iPSC colonies were individually picked and expanded for at least 5 passages before the iPS lines were confirmed positive for Tra-1–60, SSEA-4, and NANOG expression by immunofluorescence. In all fully reprogrammed iPSCs, vector-encoded transgenes were found to be silenced.

### 2.6. Spontaneous In Vitro Differentiation

Pluripotent cells’ spontaneous differentiation was induced as previously described [13] by culturing iPS cells as EBs in low attachment plates with hES media in the absence of bFGF for 7 days. EBs were transferred to 0.1% gelatin-coated dishes and cultured in differentiation medium (KO DMEM supplemented with 10% fetal bovine serum, 1 × MEM nonessential amino acids, 2 mML-glutamine, and 50 µM-mercaptoethanol (all from Thermo Scientific, Waltham, MA, USA) up to 7 days.

### 2.7. In Vitro Oligodendroglial Fate Differentiation/Proliferating OPC-Like Differentiation

We followed the published protocol [19] with slight modifications. Briefly, undifferentiated iPSCs were cultured as described to further generate EBs in low attachment plates with hES media without bFGF for 5 days, then switched to neural induction medium (NIM: DMEM/F12 supplemented with nonessential amino acids and N2) supplemented with bFGF (20 ng/mL, Sigma, St. Louis, MO, USA) and heparin (2 g/mL, Sigma, St. Louis, MO, USA) for 5 more days. Thereafter, the EBs were plated onto matrigel (Corning, NY, USA) or 20 μg/mL poly-L-ornithine (Sigma, St. Louis, MO, USA)/20 μg/mL laminin (Thermo Scientific, Waltham, MA, USA) (POL) coated 6-well plates (matrigel coated flasks were prepared by incubation with Matrigel diluted in cold DMEMF12 for 1 h at room temperature following the manufacturer’s instructions. For POL coating, flasks were incubated with 20 μg/mL poly-L-ornithine for 1 h at 37 °C or overnight at 4 °C. Flasks were washed twice with distilled water, and they were then further incubated with 20 μg/mL laminin for 2 h at 37 °C. Plates were washed 3 times with phosphate buffered saline (DPBS), before cell seeding, and cultured in NIM supplemented with bFGF, heparin and laminin (10 g/mL) for 3 additional days; the medium was then switched to NIM supplemented with retinoic acid (RA, 100 nM, Sigma, St. Louis, MO, USA), for 4 days. At this point, neuroepithelial differentiation (NE) neurorosettes were picked and plated into POL coated 6-well plates in the same medium with the addition of the sonic hedgehog (shh) agonist purmorphamine (Sigma, St. Louis, MO, USA) (1 μM), and B27 (Invitrogen, Carlsbad, CA, USA). NE colonies were detached mechanically 9 days later and cultured in suspension in 6-well Ultralow cluster plates. One day later, the medium was changed to NIM supplemented with bFGF (10 ng/mL), purmorphamine (1 μM), and B27 (Pre-OPC-like cells). After 11 days in this medium under the effect of bFGF without RA, proliferating Olig2+/NKX2.2+ oligodendrocyte progenitor cells were the majority (early OPC-like cells). Suspension cultures were switched to glial induction media (GIM; DMEM/F12, N1, B27, T3 at 60 ng/mL, biotin at 100 ng/mL, dibutyryl-cAMP at 1 μM; all from Sigma, St. Louis, MO, USA) supplemented with PDGF AA (10 ng/mL), IGF-1 (10 ng/mL), and NT3 (10 ng/mL) (Sigma, St. Louis, MO, USA) for 80–120 days for mature oligodendrocyte progenitor cells (mOPC) differentiation. During this long period of OPC suspension culture, 2/3 of the media volume was changed every 3 days. After growth-factor withdrawal from the medium for at least 3 weeks, mOPCs differentiated into O4+, MBP+ oligodendrocyte-like cells (OL).

### 2.8. Animals

For teratoma analysis, nonobese diabetic/severe combined immunodeficiency (NOD/SCID) immunodeficient mice were transferred from the Jackson Laboratories and housed and bred under the care of the animal house of the *Biobanco del Sistema Publico Andaluz* (BSSPA). Subcutaneous injection of iPS cells was performed under the ethical guidelines of Bionand Committee according to protocols approved by Andalusian Regional Animal Research Committee. After four weeks, tumors were sectioned and processed for histological analysis (hematoxylin and eosin staining).

### 2.9. Proliferation Assay

They were performed using MTT (3-(4,5-dimethylthiazol-2-yl)-2,5-diphenyltetrazolium bromide)) assay (M2128 Sigma, St. Louis, MO, USA) following the manufacturer’s instructions. MnSCs were plated at a density of 25,000 cells/24 well plate, and 570 nm absorbance was measured at the days indicated using a microplate reader. Experiments were done in triplicates.

### 2.10. qRT-PCR Assay

RNA was isolated using an RNeasy kit (Qiagen, Hilden, Germany), the following manufacturer’s protocol. First-strand cDNA was primed via oligodT oligonucleotides, and RT-PCR was performed with primer sets described at the key resource table. For quantitative RT-PCR, brilliant SYBR green (Biorad, Hercules, CA, USA) was used.

### 2.11. Immunostaining

Cells were fixed in 4% (*w*/*v*) paraformaldehyde (Sigma–Aldrich, St. Louis, MO, USA) in PBS for 15 min and blocked in 5% goat serum with 0.3% (*v*/*v*) Triton-X-100 (Sigma–Aldrich, St. Louis, MO, USA) for 1 h. Blocking buffer:PBS (1:2) was used to dilute primary antibodies (listed in the Supplementary Material). Secondary antibodies coupled to fluorescent dies (Life Technologies, Carlsbad, CA, USA) were incubated at room temperature for 45 min at 1:500. Nuclei were stained with either Hoechst 33,342 (Thermo Scientific, Waltham, MA, USA) or 4,6-diamidino-2- phenylindole (DAPI; Sigma–Aldrich, St. Louis, MO, USA). Fluorescence images were acquired using a Leica SP5 II confocal system or a Leica 6000B epifluorescence microscope.

### 2.12. Flow Cytometry

After differentiation into pre-OPC or OPC-like cells, cells were dissociated with TrypLE Select (Gibco) for 5–10 min and neutralized with DMEM containing 10% fetal bovine serum. Thereafter, the cells were fixed with 4% (*w*/*v*) paraformaldehyde for 30 min at 4 °C. Then, the fixed cells were permeabilized (Cytofix/Phosflow™ perm buffer III, Becton Dickinson, BD, Franklin Lakes, NJ, USA) and stained with the specific antibody (anti-PAX6, anti-Olig2, anti-NKX2.2, or anti-SOX9), or normal mouse IgG (Santa Cruz Biotechnology, Dallas, TX, USA) in perm/wash buffer (Becton Dickinson, Franklin Lakes, NJ, USA). Alexa Fluor 488-conjugated donkey anti-mouse IgG (Life Technologies, Carlsbad, CA, USA) was used as a secondary antibody. The stained cells were analyzed using a flow cytometer (Beckman Coulter Gallios, Brea, CA, USA). Data were analyzed using Kaluza Beckman Coulter.

### 2.13. Gene Expression Analysis

Global gene expression profiles of somatic and pluripotent cells were obtained using Illumina Human HT-12 v4.0 Expression BeadChip (San Diego, CA, USA) covering well-characterized genes, gene candidates, and splice variants with over 47,000 probes. Raw data were exported from Illumina GenomeStudio to an R session. The Limma package [20] was used to correct the background with the NormExp method [21] and to apply quantile normalization. Probes with detection *p*-value > 0.05 in at least 5% of samples were removed. The expression of those genes with more than one probe was calculated as the median value of all their probes.

Differential gene expression analysis was done applying linear models implemented in Limma [20]. For each comparison, we selected those genes with *p*-value adjusted by the false discovery rate (FDR) < 0.05. The Euclidean distance measure and the complete agglomeration method were used to perform hierarchical clustering.

Gene Ontology (GO) analysis was done using The Gene Ontology Resource (GO-enrichment analysis), which identified biologically relevant categories that are overrepresented in the input gene set [22,23]. Expression analysis systematic explorer (EASE) identifies GO categories in the input gene list that are overrepresented using jackknife iterative resampling of Fisher exact probabilities, with Bonferroni multiple testing correction. The “EASE score” is the upper bound of the distribution of Jackknife Fisher exact probabilities, which is a significance level with smaller EASE scores indicating increasing confidence in overrepresentation. We picked GO categories that have EASE scores of 0.05 or lower as significantly overrepresented. Pathway analysis was done using Ingenuity Software Knowledge Base (IKB), (Redwood City, CA, USA) to identify pathways that were significantly activated for a given input gene list. The association *p*-value between an input gene list and a known pathway was calculated using the right-tailed Fisher Exact Test. We picked pathways that had an FDR < 0.05.

Global gene expression profiles of somatic cells and iPS cells were obtained after RNA extraction and quality analysis (Bioanalyzer 2100-Agilent, Santa Clara, CA, USA). cDNA was synthesized, labeled with biotin, and hybridized with independent human Clarion-S microarrays (Affymetrix, Santa Clara, CA, USA) following Affymetrix protocol. Microarrays were scanned with an Affymetrix GeneChip Scanner 7G, and the obtained data were analyzed with Affymetrix^®^ GeneChip^®^ Command Console^®^ 2.0 software. The microarray expression dataset is publicly available at the Gene Expression Omnibus (GEO) repository. Further analyses were performed using the Transcriptome Analysis Console (TAC, Affymetrix, Santa Clara, CA, USA) v4.0 10 software and R version 3.5.0.

The accession number for the expression arrays data reported in this paper is NCBI’s Gene Expression Omnibus (GEO) repository: GSE151306 (https://www.ncbi.nlm.nih.gov/geo/query/acc.cgi?acc=GSE151306).

### 2.14. Migration Assay of Early OPC-Like Cells

Migration of early OPCs was performed using a 12-well Transwell chamber with 8 μm pore-size (Corning, NY, USA). Generated early OPCs were plated on the upper wells at 40 × 10^4^ cells/mL with OPC fasting medium (NeuroBasal medium with 100 units/mL penicillin and 100 μg/mL streptomycin; all from Thermo Scientific, Waltham, MA, USA), and 500 μL of 24 h CM recovered from either CT or SPMS-derived early OPC cells and of indicated factor: NG2 3 ng/μL (R&D Systems, Minneapolis, MN, USA), laminin 10 ug/mL (Thermo Scientific, Waltham, MA, USA), or bFGF 5 ng/mL (Thermo Scientific, Waltham, MA, USA), was added to lower well of the chamber. After 24 h of culture at 37 °C, cells on the upper surface of the membrane were removed with a cotton swab, whereas migrated cells on the lower membrane surface were fixed in 4% paraformaldehyd for 15 min and stained in 0.5% crystal violet aqueous solution (Sigma–Aldrich, St. Louis, MO, USA) in 20% methanol for 20 min, rinsed 3× with H2Odd, immersed in methanol for 15 min to solubilize the dye. The absorbance of the extracted solution was read (OD 560).

### 2.15. Conditional Medium (CM) Recovery

Early OPC or mOPC cells were cultured in fasting medium. After 24 h, CM was recovered, centrifuged at 1000× *g* for 5 min, and 0.45 μm filtered to eliminate cell debris and either used for migration assay or concentrated using 3 MW Amicon columns (Thermo Scientific, Waltham, MA, USA) (4000× *g* 30 min) for proteomic secretome analysis.

The cell pellet was processed separately for protein extraction using lysis buffer (10 mM tris, 150 mM NaCl, 10 mM EDTA, NP-40 1%, 1 mM Sodium Ortovanadate (all from Sigma–Aldrich, St. Louis, MO, USA), and a tablet of C-complete (Roche, Basel, Switzerland) protease inhibitor cocktail). Proteins were precipitated using deoxicholate (0.02%, 30 min 4 °C) and trichloroacetic acid (TCA) (Sigma–Aldrich, St. Louis, MO, USA) (10%, 18 h 4 °C), after cold acetone washing, samples were precipitated, air dried, and −80 °C frozen for further LC-MS analysis.

### 2.16. Sample Preparation for LC-MS Analysis

Samples were cleaned to remove contaminants by protein precipitation with trichloroacetic acid (TCA)/acetone and solubilized in 50 µL of 0.2% RapiGest SF (Waters, Milford, MA, USA) in 50 mM ammonium bicarbonate. Total protein content was measured using the Qubit Protein Assay Kit (Thermo Fisher Scientific, Waltham, MA, USA), and 50 µg of protein were subjected to trypsin digestion following a protocol adapted from Vowinckel et al. [24]. Briefly, protein samples were incubated with 5 mM dithiothreitol (DTT) at 60 °C for 30 min, and then with 10 mM iodoacetamide at room temperature and darkness for 30 min. Sequencing Grade Modified Trypsin (Promega, Madison, WI, USA) was added (ratio 1:40 trypsin:protein) in two steps, incubating at 37 °C for 2 h in the first step and 15 h at the second step. RapiGest was then precipitated by centrifugation after incubating with 0.5% trifluoroacetic acid (TFA) at 37 °C for 1 h. The final volume was adjusted with milliQ water and acetonitrile (ACN) to a final concentration of 0.5 µg peptide/µL, 2.25% ACN, and 0.2% TFA.

### 2.17. Creation of the Spectral Library

To build the MS/MS spectral libraries, the peptide solutions were analyzed by a shotgun data-dependent acquisition (DDA) approach by nano-LC-MS/MS. Each sample (2 μL) was separated into a nano-LC system Ekspert nLC415 (Eksigent, Dublin, CA, USA) using an Acclaim PepMap C18 column (75 μm × 25 cm, 3 µm, 100 Å) (Thermo Fisher Scientific, Waltham, MA, USA) at a flow rate of 300 nl/min. Water and ACN, both containing 0.1% formic acid, were used as solvents A and B, respectively. The gradient run consisted of 5% to 30% B in 120 min, 10 min at 90% B, and finally 20 min at 5% B for column equilibration, in a total run time of 150 min.

As the peptides eluted, they were directly injected into a hybrid quadrupole-Time of Flight (TOF) mass spectrometer Triple TOF 5600+ (Sciex, Redwood City, CA, USA) operated with a ‘top 65′ data-dependent acquisition system using positive ion mode. A NanoSpray III ESI source (Sciex, Redwood City, CA, USA) was used for the interface between nLC and MS, applying a 2600 V voltage. The acquisition mode consisted of a 250 ms survey MS scan from 350 to 1250 *m*/*z*, followed by an MS/MS scan from 230 to 1700 *m*/*z* (60 ms acquisition time, rolling collision energy) of the top 65 precursor ions from the survey scan, this making a total cycle time of 4.2 s. The fragmented precursors were then added to a dynamic exclusion list for 15 s; any singly charged ions were excluded from the MS/MS analysis.

The peptide and protein identifications were performed using Protein Pilot software (version 5.0.1, Sciex, Redwood City, CA, USA) with a human Swiss-Prot concatenated target-reverse decoy database, specifying iodoacetamide as Cys alkylation. The false discovery rate (FDR) was set to 0.01 for both peptides and proteins. The MS/MS spectra of the identified peptides were then used to generate the spectral library for sequential windowed acquisition of all theoretical fragment ion (SWATH) peak extraction using the add-in for PeakView Software (version 2.1, Sciex, Redwood City, CA, USA) MS/MSALL with SWATH Acquisition MicroApp (version 2.0, Sciex, Redwood City, CA, USA). Peptides with a confidence score above 99%, as obtained from the Protein Pilot database search, were included in the spectral library.

### 2.18. Relative Quantification by SWATH Acquisition

The samples were then analyzed using a DIA method. Each sample (2 μL) was analyzed using the LC-MS equipment and LC gradient described above for building the spectral library, but using a SWATH-MS acquisition method. The method consisted of repeating a cycle consisting of the acquisition of 50 TOF MS/MS scans of overlapping sequential precursor isolation windows of variable width (1 *m*/*z* overlap) covering the 350 to 1250 *m*/*z* mass range (350 to 1200 *m*/*z* for the adipose tissue sample set), with a previous MS scan for each cycle. The accumulation time was 50 ms for the MS scan (from 350 to 1250 *m*/*z*) and 100 ms for the product ion scan (230 to 1500 *m*/*z*, high sensitivity mode), thus making a 5.1 s total cycle time. For each sample set, the width of the 50 variable windows was optimized according to the ion density found on DDA runs using a SWATH variable window calculator worksheet from Sciex (Redwood City, CA, USA).

### 2.19. Proteomic Data Analysis

The targeted data extraction of the SWATH runs was performed using the add-in for PeakView Software (version 2.1, Sciex, Redwood City, CA, USA) mass spectometry of all possible candidates (MS/MSALL) with SWATH Acquisition MicroApp (version 2.0, Sciex, Redwood City, CA, USA). This application processed the data using the spectral library created from the shotgun data, extracting and integrating the fragment ion chromatograms from the SWATH runs. Up to ten peptides per protein and seven fragments per peptide were selected, based on signal intensity; any shared and modified peptides were excluded from the extraction. Five-minute windows and 20 ppm widths were used for extracting the ion chromatograms, and SWATH quantitation was attempted for all proteins in the ion library that were identified by ProteinPilot with an FDR below 1%. The retention times from the peptides that were selected for each protein were realigned in each run according to iRT peptides (Biognosys AG, Schlieren/Zürich, Switzerland) spiked in each sample and eluting along the whole time axis.

For testing for differential protein abundance between the three groups, MarkerView (v1.2.1, Sciex, Redwood City, CA, USA) was used. This application first normalizes the data across samples and then computes the protein fold changes between the experimental groups. The output of this determination is a fold change and a *p*-value for each protein.

## 3. Results

### 3.1. Successful Derivation Generation of iPS Cell Lines from SPMS Donors

**Figure 1:**
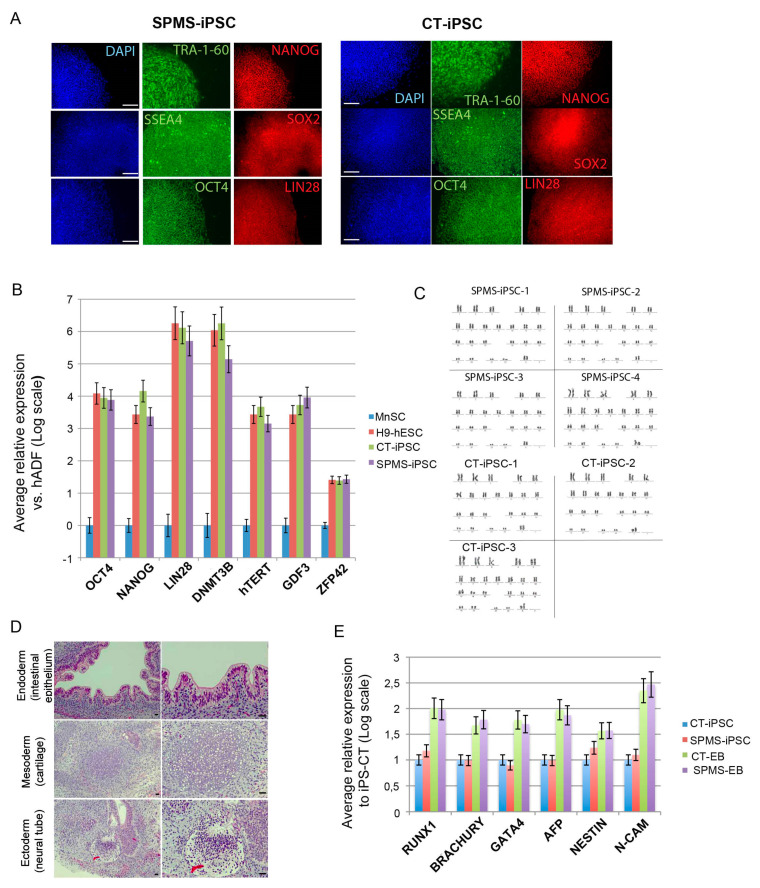
Successful generation of induced pluripotent stem (iPS) cell lines from secondary progressive multiple sclerosis (SPMS) donors. (**A**) Representative immunofluorescence analysis image of pluripotent markers on iPSCs-multiple sclerosis (MS) and iPSC-control (CT) colonies shows similar staining pattern (Scale bar = 100 μm). (**B**) qRT-PCR for genes characteristic of pluripotent cells was performed as indicated with mRNA collected from source menstrual blood-derived stromal cells (MnSCs), H9- human embryonic stem cells (hESCs), three reprogrammed CT-iPSCs, and four SPMS-iPSCs. Values indicate average relative expression of the specific gene normalized to glyceraldehyde 3-phosphate dehydrogenase (GAPDH)/Actin relative to MnSC expression, which was arbitrarily assigned a value of zero on a logarithmic scale. Data correspond to the average of 3 independent experiments done in triplicate. (**C**) High-resolution G-banded karyotypes. Representative four clones of fully reprogrammed SPMS-iPSCs and three CT-iPSCs showing normal karyotype. (**D**) Representative hematoxylin and eosin-stained sections of matured SPMS-iPSC-derived teratomas exhibiting characteristic structure of intestinal epithelium (endoderm), cartilage (mesoderm), and neural epithelium (ectoderm) (Scale bar = 50 μm). (**E**) qRT-PCR data showing upregulation of differentiation markers GATA4 and AFP (endoderm), RUNX1, and BRACHURY (mesoderm) and NCAM and Nestin (ectoderm) at day 10 of in vitro spontaneous differentiation protocol. Embryoid bodies (EBs) were derived from SPMS- and CT-iPSCs. Average folding change expression values ± SEM (relative to undifferentiated iPSCs) are represented (logarithmic scale).

Menstrual blood-derived stromal cells (MnSCs) were established from four secondary progressive multiple sclerosis (SPMS)-affected donors and three control (CT) healthy donors and were used to derive iPS cell lines (iPSC-MS-01 to -04 and iPSC-CT-01 to -03) through retroviral-driven overexpression of the three reprogramming factors: OCT4, SOX2 and KLF4 [17,25,26]. Three to four weeks after transduction, TRA-1-60+ colonies were picked, expanded, and characterized by immunofluorescence for pluripotency markers after at least 10 expansion passages from initial colony appearance (Figure 1A).

Gene expression analysis by quantitative PCR confirmed that all four SPMS-iPS cell lines presented a pluripotent compatible profile, similar to control iPSCs (CT-iPSC) and to the reference H9-hES cell line as compared to parental somatic cells (Figure 1B). We did not find any detectable expression of exogenous genes in the iPS cell lines indicating retroviral silencing. All SPMS-iPS cell lines showed a normal G-banded karyotype (Figure 1C) and three germ layers’ differentiation capacity, both in vivo, using teratoma assay (Figure 1D), and in vitro, using spontaneous embryoid body (EB) differentiation (Figure 1E).

Results indicated successful and uniform reprogramming across all SPMS- and control-derived iPS cell lines.

### 3.2. Efficient Differentiation of SPMS-iPS Cell Lines into Oligo Progenitor Cell Fate

We next assessed the differentiation potential towards the oligodendrocyte progenitor (OPC) lineage fate.

**Figure 2:**
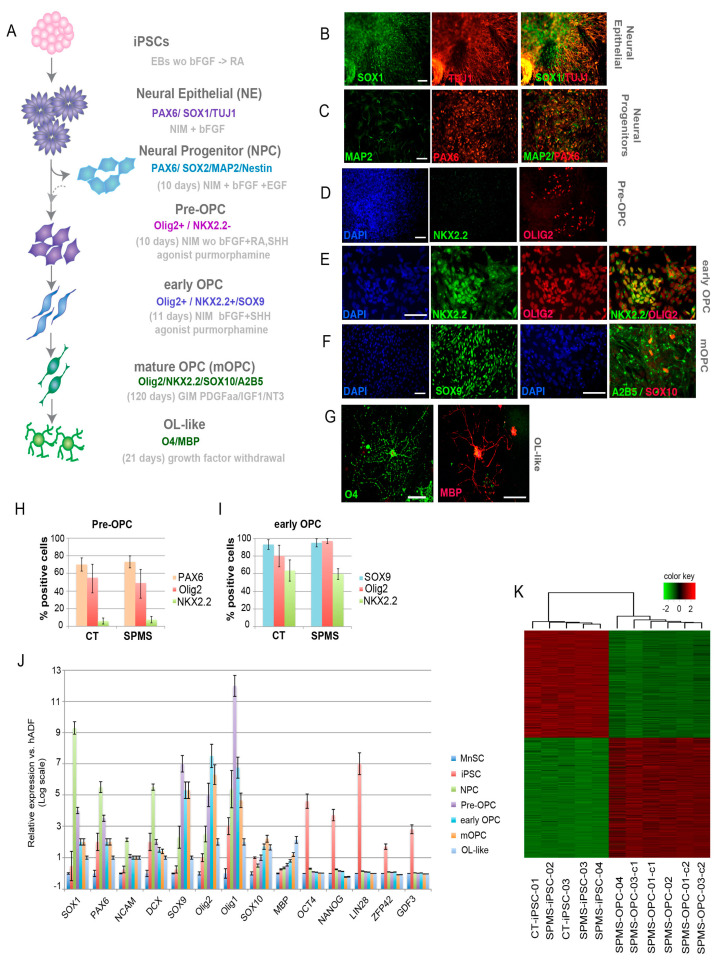
Efficient differentiation of SPMS-iPS cell lines into oligoprogenitor cell fate. (**A**) Schematic protocol for differentiation of hiPSCs into OPC-like cells. Embryoid bodies (EBs) were differentiated from hiPSCs and then differentiated as neuroepithelial (NE) cells in neural induction media (NIM) with bFGF as described in experimental procedures. (**B**–**F**) Representative immunofluorescence images of SPMS-iPSCs at each of the differentiation stages underlined: (**B**) SPMS-iPSC-derived NE cells at this stage expressing the neuroepithelial markers PAX6 and TUJ1; (**C**) When NE cells are subjected to neural progenitor cell media containing basic fibroblast growth factor (bFGF) and EGF they express PAX6 and MAP2 markers; (**D**) When NE cells are cultured in the absence of bFGF and in the presence of RA and SHH agonist purmorphamine they express the pre-oligo progenitor (pre-OPC) marker Olig2 but not NKX2.2; (**E**) After further culture with bFGF and purmorphamine without RA, proliferative OPC-like cells show Olig2 and NKX2.2. (**F**) Culture of OPC-like cells in glial induction media (GIM) gives rise to mOPC with SOX9-, A2B5-, and SOX10-positive labeling. (**G**) After growth factor withdrawal, they gave rise to oligodendrocyte-like (OL) cells expressing O2 and MBP markers. (**H**,**I**). Flow cytometric analysis at pre-OPC (**H**) and early OPC-like (**I**) stages of CT- and SPMS-derived samples, showing the percentage of positive cells for each selected marker. Data are expressed as mean ± SEM (*n* = 9) with cells from 3 different donor iPSCs. (**J**) qRT-PCR for gene characteristics of oligodendroglial fate specification and to check pluripotency repression was performed as indicated on mRNA collected from SPMS-iPS derived cells at indicated stages. Values indicate average relative expression of the specific gene normalized to GAPDH/Actin relative to MnSC expression, which was arbitrarily assigned a value of zero on a logarithmic scale. Data correspond to the average of four independent experiments (four SPMS-iPS cell lines from four donors) done in triplicate. (**K**) Correlation heatmap showing the clustering of iPSCs and SPMS early OPC-like lines using array-based RNA expression data. Euclidean distance and the complete agglomeration method were used to compute the heatmap dendrogram. Correlation was computed with Pearson’s method.

We performed iPSC differentiation of both SPMS-iPSCs and CT-iPSCs following the six-stages protocol published by Goldman’s laboratory [19] with slight modifications, as detailed in the Materials and Methods and schematized in Figure 2A. Briefly, iPSCs were manually picked and plated into nonadherent conditions to form embryoid bodies (EBs) in the absence of bFGF to trigger pluripotent genes silencing. After EBs plating, retinoic acid (RA) was added, as it has been described to be critical for oligodendrocyte specification [16]. At this point, rosette-like colonies were evident and expressed neural epithelial (NE) markers (PAX6, SOX1, and Tuj1; 63 ± 7.3% of the colonies in SPMS-iPSCs and 60 ± 8.1% in CT-iPSCs; Figure 2B and Figure S1A). Mechanically detached NE colonies could be cultured to form neural progenitors expressing PAX6, SOX2, Nestin, and MAP2 (Figure 2C and Figure S1B), or biased to the glial progenitor lineage through the addition of the sonic hedgehog (SHH) agonist purmorphamine [19] to generate first pre-oligoprogenitor cells (pre-OPC) expressing Olig2 but with low NKX2.2 (Figure 2D and Figure S1C). Then, after RA removal, proliferating early oligodendrocyte progenitor-like cells (OPC-like) expressed Olig2, Sox9, and NKX2.2 (Figure 2E and Figure S1D). Long culture under glial induction medium (GIM) with PDGF AA, IGF-1, and NT3, led to the formation of mature OPC (mOPC), upregulating SOX10 and A2B5 (Figure 2F and Figure S1E), which, after culture with a reduction in mitogens, allowed terminal differentiation into oligodendrocyte-like (OL) O4- and MBP-positive cells (Figure 2G and Figure S1D).

Flow cytometry analysis (Figure 2H,I) and quantitative PCR (Figure 2J) at different differentiation stages and high-throughput, array-based expression patterns of derived early OPC-like cells (Figure 2K) confirmed immunofluorescence data and showed that both SPMS and CT-iPSCs perform similar OPC-like lineage differentiation efficiencies with a minimal representation of astrocyte and neuronal lineage markers (Figure S2A–D). Our results with SPMS-iPSCs agree with previously published data indicating iPSCs derived from PPMS donors can efficiently generate myelinating oligodendrocytes [16].

### 3.3. Differences in SPMS-Derived Cells Arise after Differentiation into OPC-Like Cells

**Figure 3:**
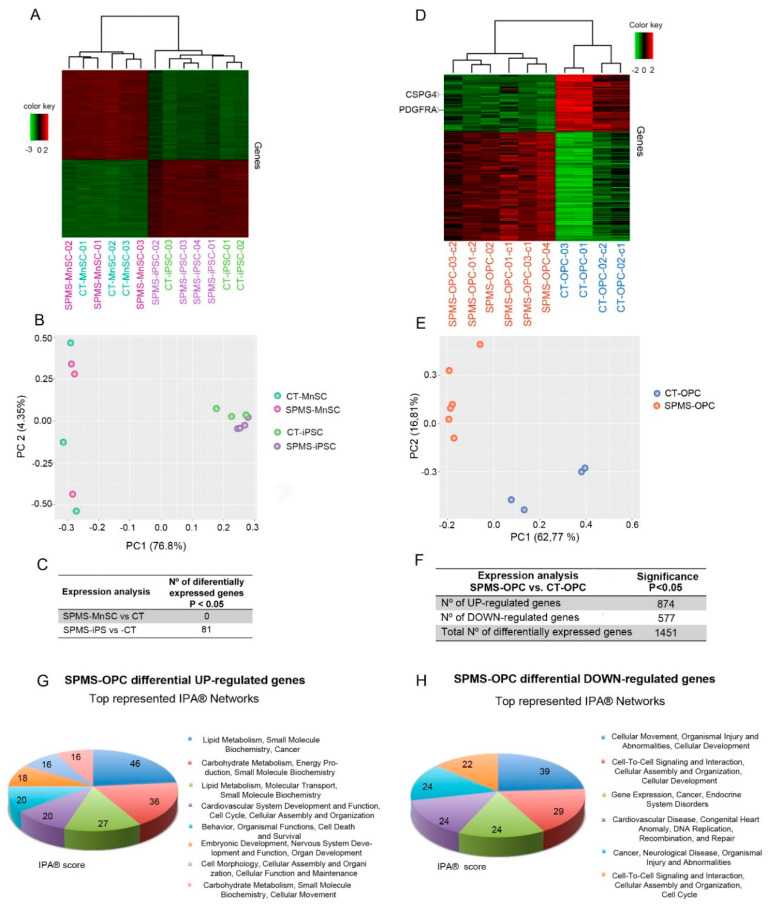
Transcriptomic differences in SPMS-derived cells arise after differentiation into early oligoprogenitor cell (OPC)-like cells. (**A**) Correlation heatmap showing the clustering of SPMS- and CT-derived somatic cell lines and isogenic generated iPSCs using high-throughput array-based expression data. The Euclidean distance and complete agglomeration method were used to compute the heatmap dendrogram. Correlation was computed with Pearson’s method. (**B**) Principal component analysis (PCA) using expression data, as in Figure 3A. (**C**) Differentially regulated genes between SPMS and CT groups using somatic or pluripotent cells (*p*-value adjusted by false discovery rate (FDR) < 0.05). (**D**) Correlation heatmap showing the clustering of SPMS- and CT-derived early OPC-like cells using RNA expression (array-based) data, as in Figure 3A. (**E**) PCA analysis using expression data of early OPC-like cells. (**F**) Differentially regulated genes between SPMS- and CT-derived OPC-like cells (*p*-value < 0.05). (**G**,**H**) Identification of the top represented significantly activated networks using Ingenuity^®^ Pathway Analysis (IPA^®^) based on upregulated (**G**) or downregulated (**H**) genes in SPMS-derived early OPC-like cells.

We performed whole-transcriptome profiling on each cohort of cells derived from each multiple sclerosis (SPMS) or control (CT) donors, including MnSCs, iPSCs, and proliferating early OPC-like cells. As expected, when we compared CT- and SPMS-derived MnSCs, they did not show significant differentially regulated genes. We obtained a similar result using their genetically matched generated iPSCs, with only 81 differentially regulated genes and scrambled both hierarchical and principal component analysis (PCA) clustering (Figure 3A–C). However, unsupervised hierarchical clustering and PCA revealed a separated grouping of SPMS- from CT-derived early OPC-like cells (SPMS-OPC and CT-OPC), and they presented 1451 differentially regulated genes (Figure 3D–F and Table S1).

These results indicate that, although somatic and pluripotent cells from SPMS-affected and healthy donors were generally indistinguishable, differences arose when they differentiated towards the oligodendroglial fate. OPCs have an important role in myelin repair and have been suggested to be affected in the progressive form of multiple sclerosis [27,28,29]; thus, these results support donor-iPSC derivation and OPC-like differentiation constituting an appropriate cell model for the secondary progressive form of MS.

Although, as expected, a number of OPC characteristic genes appeared upregulated in all CT and SPMS-derived early OPC-like cells with respect to iPSCs (notably NKX2.2, LRRN1, TRAF4, SOX10, SOX6, Olig2, POU3F1, MMP15, IGSF21, NEV4) as a result of the successful and similar differentiation process, we identified NG2/GSPG4 and PDGFRα among genes specifically downregulated in SPMS-OPC that were confirmed by immunofluorescence (Figure S2E). These are surface proteins that have been widely used as proliferating OPC markers [30]. NG2/CSPG4 has been supposed to be involved in the PDGF signaling in OPCs, acting as co-receptor of PDGFRα [31,32]. Although the function of NG2/CSPG4 in OPCs is still under study, it has been described to play an essential role in cell proliferation, migration, cytoskeleton organization and neuro-modulation [33,34,35,36,37,38,39,40].

Differentially expressed genes were analyzed using Ingenuity Pathway Analysis (IPA^®^) (Redwood City, CA, USA) to analyze top represented network profiles and canonical pathways. We found that SPMS-derived early OPC-like cells upregulate genes involved, among other signaling networks, in lipid and carbohydrate metabolism and small molecule biochemistry (Figure 3G), while downregulated genes pointed to alterations in pathways involved in cellular movement, cell–matrix and cell-to-cell signaling and interaction (Figure 3H).

IPA^®^ canonical pathway and Gene Ontology (GO) biological analysis of differentially expressed genes between SPMS- and CT-derived early OPC-like cells included Integrin and Notch Signaling, or dopamine and serotonin receptor signaling (Figure S3A), and biological processes related to cellular motility (Figure S3B). Integrin and Notch signaling has been evidenced as crucial for OPC identity and function [41,42,43], further supporting the use of SPMS cell modeling through iPSC derivation.

### 3.4. Analysis of Proteins Differentially Secreted by SPMS-Derived OPC-Like Cells

**Figure 4:**
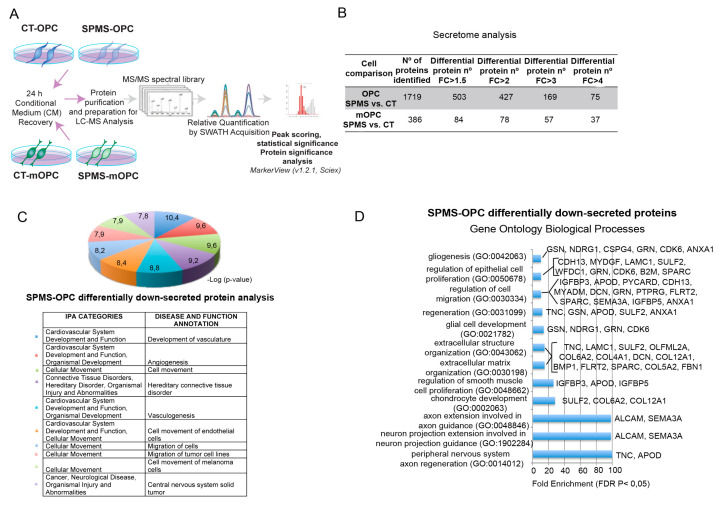
Analysis of proteins differentially secreted by SPMS-derived OPC-like cells. (**A**) Schematic protocol for supernatant recovery and secretome analysis. SPMS- and CT-derived early OPC-like and mature OPC (mOPC) cells were cultured in fasting media (see methods) for 24 h. Incubated media were recovered, and proteins were purified, prepared for liquid chromatography–mass spectrometry (LC-MS), and used to create a MS/MS spectral library using a SWATH-MS acquisition method for relative quantification. Data analysis was performed using the add-in for PeakView Software, and for testing differential protein abundance between the selected groups, MarkerView (v1.2.1, Sciex) was used. (**B**) Secretome analysis of SPMS and CT-derived early OPC-like and mOPC cells. Number of proteins differentially represented (*p* < 0.05) between SPMS and CT samples with a folding change (FC), over 1.5, 2, 3, or 4. (**C**,**D**). Identification of the top represented significant disease and function categories using Ingenuity^®^ Pathway Analysis (IPA^®^) (**C**) or annotated Gene Ontology (GO) Biological Processes (**D**) based on downsecreted proteins in SPMS-derived early OPC-like cells.

Our gene ontology and pathway analysis of specifically regulated genes in SPMS-derived early OPC-like cells pointed to differences in cell–cell and cell–matrix communication, functions that are highly influenced by secreted proteins and factors. Thus, we aimed to characterize the secretome of SPMS- and CT-derived early OPC-like and mOPC cells. We performed a nontargeted systematic proteomic-based quantitative analysis to compare protein secretion patterns of collected conditioned media (CM) from each cell group (schematized in Figure 4A).

SWATH-Mass Spectrometry (SWATH-MS) analysis allowed the relative quantification of 1719 secreted proteins in early OPC-like cells: 503 of them were differentially secreted between affected and nonaffected groups (*p* < 0.05), with a folding change (FC) > 1.5; 427 proteins with FC > 2; 169 proteins with FC > 3; and 75 with FC > 4 (Figure 4B and Table S2).

Similar analyses with mOPCs CM allowed the relative quantification of 386 secreted proteins: 84 of them were significantly different between multiple sclerosis and control groups (*p* < 0.05; FC > 1.5; Figure 4B and Table S3).

To gain further insight into the biological functions of differentially secreted proteins in proliferating early OPC-like cells, the IPA^®^ software annotation term enrichment tool was used (Figure 4C). This showed SPMS-derived early OPC-like conditioned media was enriched in proteins related to cytoskeleton dynamics, axon and neurites organization, or cell–cell contact (Figure S4A), while proteins undersecreted by SPMS-OPC were related to cell movement and migration (Figure 4C). Gene Ontology (GO) analysis showed biological processes as neuron projections guidance, extracellular matrix organization, cell migration, or cell proliferation (Figure 4D).

The same GO analysis with identified proteins secreted by mOPC cells, showed proteins underrepresented in SPMS-derived samples were associated with extracellular matrix organization, cell adhesion, or cell differentiation (Figure S5).

### 3.5. SPMS-OPC Secretome Shows Deficient In Vitro Early-OPC Migration Stimulation

**Figure 5:**
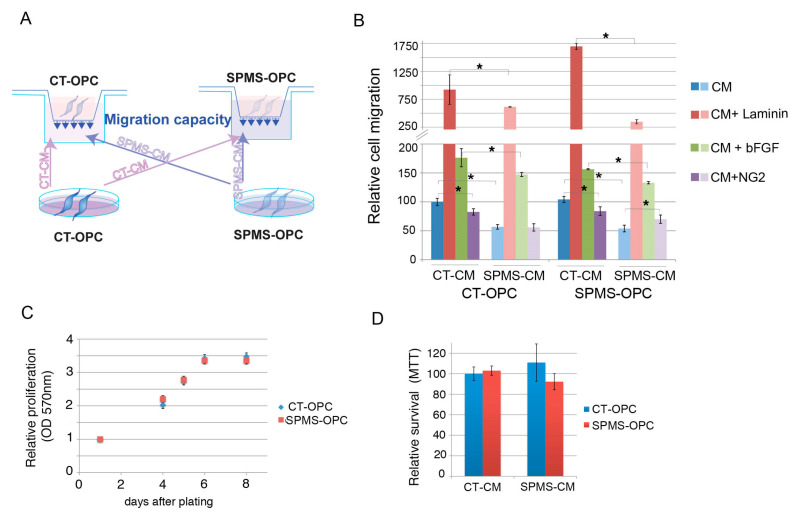
Progressive SPMS-OPC secretome shows deficient in vitro OPC migration stimulation. (**A**) Schematic protocol of migration assay. SPMS- and CT-derived early OPC-like cells were culture in fasting media, and conditional media (CM) were recovered after 24 h and added to the lower well of transwell chambers. SPMS- and CT-derived early OPC-like cells were plated on upper wells of transwell chambers, and 24 h later, migrated cells on the lower membrane surface were fixed, crystal violet stained, and quantified by optical density (OD 540) values of stained cell extracts. (**B**) Quantification of cell migration (OD 540) under CT- or SPMS-derived CM with indicated stimulating factors (laminin, bFGF, or NG2) relative to the CT-OPC-like sample under CT CM stimulation. Error bars represent means ± SEM (*n* = 6 independent experiments with early OPC-like cell lines from four different SPMS and three CT donors) * *p* ≤ 0.05 by unpaired Student’s *t*-test. (**C**) SPMS- and CT-derived early OPC-like cells present similar proliferation rates. A total of 25,000 OPC-like cells were cultured with NIM/bFGF/purmorphamine for proliferation/viability MTT (3-(4,5-dimethylthiazol-2-yl)-2,5-diphenyltetrazolium bromide) assay. Cells were recovered 1, 4, 5, 6, and 8 days after and measured at 570 nm absorbance, reflecting the number of viable cells (MTT assay). Average relative absorbance ± SEM (relative to day 1) is represented (*n* = 3 independent experiments with early OPC-like cell lines from four different SPMS and three CT donors). (**D**) CT and SPMS-derived OPC-like cells were cultured for 24 h in CM as in Figure 5A, and after 48 h, the number of viable cells (MTT assay) was measured. Average relative absorbance ± SEM is represented (*n* = 3 independent experiments with OPC-like cell lines from four different SPMS and three CT donors).

Our secretome analysis suggested abnormal representation of proteins related to cell migration, movement, and cell–matrix interaction. We, thus, interrogated the capacity of early OPC-like cells to migrate when we used the CM secreted by either CT or SPMS-OPC as migration stimulus by using a transwell chamber assay (schematized in Figure 5A).

We found both CT- and SPMS-derived early OPC-like cells present similar cell migration capacity in the presence of CT-OPC CM, indicating SPMS-derived early OPC-like cells have full migration capacity; however, under SPMS-OPC CM, both cell groups showed more than 45% reduction in cell migration, indicating a deficient capacity of the SPMS-OPC secretome in activating in vitro OPC migration (Figure 5B).

We next analyzed whether early OPC-like cells responded to known powerful migration stimuli, such as the extracellular matrix component laminin or the mitogen factor bFGF, and whether they can reverse deficiencies in the SPMS OPC secretome.

We found both stimuli highly increased SPMS and CT early OPC-like cells migration when combined with both SPMS- and CT-generated secretomes, indicating they overcome deficiencies in SPMS-OPC CM composition (Figure 5B and Figure S6).

To further investigate more specific factors to compensate SPMS-OPC CM impairment in cell migration stimulation, we focused on neuron-glial antigen 2 (NG2), as this factor has been described to regulate cell migration and survival in the central nervous system [36], and it is both downregulated and downsecreted in SPMS-derived OPC-like cells. When combined with CT CM, NG2 reduced the cell migration in both CT and SPMS OPCs; however, when we added NG2 to SPMS CM, there was an increase in SPMS-OPC migration (Figure 5B and Figure S6), supporting previous works showing NG2 has a pro-migratory effect [33]. Our results indicated there are specific components in SPMS-OPC-derived CM that can be exogenously added to compensate for its deficient effect on in vitro OPC migration stimulation.

We found a similar basal cell proliferation rate in SPMS- and CT-derived early OPCs (Figure 5C), and there were no differences in cell survival after either SPMS- or CT-derived OPC CM (Figure 5D), indicating differences found in cell migration stimulation are not due to reduced cell viability.

Together, our SPMS-OPC-like cell model and secretome analysis provide a valuable list of proteins that can be involved in secondary progressive MS pathology. Our data suggest that impaired early OPC communication for cell migration can potentially contribute to deficient OPC localization and differentiation to rescue demyelinated areas.

## 4. Discussion

Our results showed that SPMS cellular models can be generated using iPSC technology, which supports similar works indicating iPSC lines of patients with PPMS can efficiently differentiate to OPC and mature myelin-forming OLs [16] and to neural progenitor cells [14] according to general cell fate markers. This allowed the exploration of the functional characteristics in specific cell types potentially altered in PPMS, as studies describing neural progenitor cells were less able to provide neuroprotection to myelin injury or support OPC differentiation in vitro [14] partially due to senescence activation in neural progenitor cells (NPCs) [15].

However, our data indicated that even if proliferating early OPC-like cells are efficiently generated from both CT and SPMS-derived iPSC, they show distinguishable expression profiles, thus clustering separately, and they differently secrete a number of proteins.

Functional annotation analysis of differentially represented proteins in SPMS-OPC CM allowed us to identify extracellular matrix, cell adhesion, and migration signaling categories. We further confirmed SPMS early OPCs present similar proliferation and migration capacities compared to CT-derived cells, but they fail in the signaling capacity of their CM to stimulate in vitro cell migration. Of note, deficient migration stimulation can be reversed by exogenous addition of known activators of cell migration to SPMS-OPC CM, such as the extracellular matrix laminin or the growth factor bFGF, and partially by the specific protein downsecreted in the SPMS OPC secretome, NG2/CSPG4. This is especially relevant, as extrinsic inhibition of differentiation in the lesion environment is considered the main mechanism of remyelination failure and a body of evidence supports that extracellular matrix (ECM) changes, including the proteases that shape the ECM, impact the outcome of repair after demyelination [44]. Thus, differences found in ECM and extracellular signaling molecules secreted by the OPC population in SPMS can contribute to creating an altered environment for remyelination.

The extracellular domain of NG2/CSPG4 can be proteolytically released (shed) from the cell surface both in vitro and in vivo [45,46]. Soluble NG2/CSPG4 released from OPC cell surfaces modulates their migration [47,48] and regulates their cell polarity with a direct effect on cell migration [49]. We showed here that the addition of NG2 recombinant protein to the CM produced by control OPC-like cells causes an inhibitory effect on cell migration but an activating effect when NG2 is combined with SPMS-derived CM, suggesting the fine regulation of NG2 concentration on migration signaling. It is possible that NG2/CSPG4 stimulation of cell migration works in a concentration range and depends on other factors present in the media, as CSPG4-mediated molecular events are articulated through the interaction with more than 40 putative ligands [50]. Additionally, it has been described that NG2/CSPG4 posttranslational modification domains are crucial for proper signaling and cell function [50]. We cannot discard that the presence of plasma membrane NG2 at normal levels is needed, as it has been described to be a core organizer of Rho GTPase activity and localization in the cell, controlling OPC polarity and directional migration [49].

Expression of NG2 underlies a finely balanced regulation; it is influenced by extracellular (such as inflammation or hypoxia) and intracellular factors (methyltransferases, miRNAs, or transcription factors), and it is dependent on specific cell functions in different tissues [51]. However, which factors are involved in this regulation and how they interact with each other remains elusive, and further studies are needed to unravel NG2-specific modifications and interactions in OPCs. Our work contributes an SPMS cell model using iPSCs to further study such modifications with observed defective migration stimulation and other potential alterations in affected cells.

OPC differentiation in vivo is limited and mostly arrested, resulting in chronic demyelination [27,28,29,52]. Studies have revealed the presence of undifferentiated OPCs in progressive MS lesions [29,53]. This finding highlights an obstacle in the process of remyelination: the inability of OPCs to mature into myelin-producing OLs. Our results support that progressive multiple sclerosis cellular models can be generated using iPSC technology. Differently to in vivo lesion observations, iPSC lines of patients with SPMS can efficiently differentiate to OPC, although with a different expression profile, and to mature myelin-forming OLs, which agrees with efficient PPMS-iPSC oligodendroglial [16] and neural progenitor differentiation data [14]. There are several factors that might be responsible for this phenomenon [7,54]. An absence of any of the necessary signaling molecules might inhibit endogenous differentiation. This underscores the importance of identifying the different cell types responsible for the production of molecules required for proper activation, proliferation, migration, and differentiation. In this sense, endogenous and transplanted neural progenitor cells (NPCs) have been found to be capable of secreting factors that are anti-inflammatory factors and support remyelination [55,56,57,58]. Recent evidence shows that PPMS-iPS-derived NPCs activate senescence signaling, thus inhibiting oligodendrocyte differentiation [15].

Similarly, defective OPC migration could result in missed differentiation cues at the lesion site. Our results reinforce the notion of a component of progressive MS based on a signaling defect in remyelination and suggest early OPC migration capacity and stimulation as potential target to restore remyelination and neurological function.

Interestingly, the latest single-cell transcriptomic data [59] reveal heterogeneity in oligodendrocyte lineage clusters in human postmortem MS samples. Data also show that oligodendrocytes in ’normal appearing white matter’ (NAWM) regions present different molecular signatures to control individuals, and they differ from a specific oligodendrocyte lineage signature at lesion sites as well. The authors conclude that oligodendrocytes that appear morphologically normal in individuals affected by MS are affected by disease and are in an altered state. In addition, Frisen and colleagues [60] showed, using a 14C-based cellular dating technique in autopsy samples, that NAWM was affected in MS postmortem tissue and revealed an unexpected heterogeneity of patients with respect to the response of OPCs to the disease. They suggest that lesions thought to exhibit incomplete myelin repair (shadow plaques) are not generally generated from newly recruited OPCs in MS but from preexisting mature oligodendrocytes. It is possible that the scenario proposed by these authors is generated or affected by defective OPC migration signaling in MS individuals, forcing the lesion site to use surviving oligodendrocytes for remyelination. Additionally, differences in ECM and extracellular signaling molecules secreted by the OPC population in MS could affect abnormal responses to demyelination. This model would also support the use of iPS cell modeling to finely investigate the differentiation potential of SPMS-derived cells towards specific oligodendrocyte subpopulations heterogeneously observed in the mentioned postmortem samples.

The MS lesion is a complex environment, whereby a number of extracellular signals act to prevent successful remyelination. This methodology excludes the role of the lesion environment and focuses on cell-intrinsic factors that may inherently be different between MS patients and nonaffected individuals, supporting recent studies in postmortem tissue transcriptomic and genomic analysis [59,60]. Our study is limited as it focuses on proliferating early OPCs, although results agree with other studies carried out using an NPC population [14,15], which actually supports the intrinsic cell alteration in SPMS shown here. We are aware of the limitations in cell differentiation protocols and cell identity characterization at the population level in our study, even more in a disease with different cell components involved. Future experiments using single-cell transcriptome analyses would overcome such limitations from mixed cell population analyses and would contribute to defining disease progression at the cellular level. To mention, although a majority of RRMS patients evolve to SPMS within 10–25 years, in this study, we focused exclusively on SPMS samples, and future studies will be required to determine whether our results are features of progressive disease or general features of MS. A bonafide iPSC-derived model to recapitulate disease progression is an ambitious goal for the scientific community; future studies will need combinations of all different cell types involved in the disease, 3D interaction models, and functional simulation.

## Figures and Tables

**Table 1:** Somatic cell donor information.

Line	Cell Type	Gender	Age	Disease	Clinical Diagnosis SPMS
SPMS-01	MnSC	Female	39	SPMS	2 years
SPMS-02	MnSC	Female	43	SPMS	8 years
SPMS-03	MnSC	Female	42	SPMS	5 years
SPMS-04	MnSC	Female	40	SPMS	3 years
CT-01	MnSC	Female	37	No	n/a
CT-02	MnSC	Female	39	No	n/a
CT-03	MnSC	Female	41	No	n/a

SPMS: Seconcary Progressive Multiple Sclerosis; CT: Control (nonaffected); MnSC: Menstrual blood-derived stromal cells; n/a: not applicable.

## Supplementary Materials

The following are available online at https://www.mdpi.com/2073-4409/9/8/1803/s1, Figure S1: Efficient differentiation of CT-iPS cell lines into oligodendrocyte progenitor-like cell fate (related to Figure 2), Figure S2: Analysis of cell markers in early OPC-like and mOPC populations at the time of transcriptomic/secretome analysis (related to Figure 2), Figure S3: Functional annotation of SPMS-OPC differentially regulated genes (related to Figure 3), Figure S4: Identification of the top significantly represented disease-and-function categories of secreted proteins of SPMS-derived early OPC-like cells (related to Figure 4), Figure S5: Identification of the top significantly represented disease-and-function categories of secreted proteins of SPMS-derived mOPC cells (related to Figure 4), Figure S6: OPC in vitro cell migration after laminin, bFGF and NG2 stimulation (related to Figure 5) performed in basal media, Supplementary Figure S7: Graphical abstract with main results of the manuscript, Supplemental datasheet-Table S1: Differential gene expression analysis of SPMS- and CT-derived proliferating early OPC-like cells (related to Figure 3), Supplemental datasheet-Table S2: List of proteins differentially represented in conditional media from SPMS- and CT-derived proliferating early OPC-like cells (related to Figure 4), Supplemental datasheet-Table S3: List of proteins differentially represented in conditional media from SPMS- and CT-derived mOPC cells (related to Figure 4) and Supplemental Resources Table.
