# Supplementary material for: iPS-Derived Early Oligodendrocyte Progenitor Cells from SPMS Patients Reveal Deficient In Vitro Cell Migration Stimulation
Source: Cells. 2020 Jul 29;9(8):1803. doi: 10.3390/cells9081803 (PMC7463559; doi:10.3390/cells9081803)
Supplement: Supplementary file 1 [file cells-09-01803-s001.zip › cells-855634 supplementary final/supplementary proof done.docx]

Article

iPS-Derived Early Oligodendrocyte Progenitor Cells from SPMS Patients Reveal Deficient In Vitro Cell Migration Stimulation

Lidia Lopez-Caraballo ^1^, Jordi Martorell-Marugan ^2,3^, Pedro Carmona-Sáez ^2,4^ and
Elena Gonzalez-Munoz ^1,5,6,^*

^1^ Laboratory of Cell Reprogramming (LARCEL), Andalusian Centre for Nanomedicine and
Biotechnology-BIONAND, 29590 Málaga, Spain

^2^ Bioinformatics Unit. GENYO, Centre for Genomics and Oncological Research: Pfizer/University of Granada/Andalusian Regional Government, PTS Granada, E-18016 Granada, Spain; jordi.martorell@genyo.es (J.M.-M.); pedro.carmona@genyo.es (P.C.-S.)

^3^ Atrys Health, 08025 Barcelona, Spain

^4^ Department of Statistics. University of Granada, 18071 Granada, Spain

^5^ Department of Cell Biology, Genetics and Physiology, University of Málaga, 29071 Málaga, Spain

^6^ Networking Research Center on Bioengineering, Biomaterials and Nanomedicine, (CIBER-BBN), 29071 Málaga, Spain

***** Correspondence: egonmu@uma.es; Tel.: +34952367616

Received: 18 June 2020; Accepted: 28 July 2020; Published: date

Supplementary Figure S1 is related to Figure 2

Supplementary Figure S2 is related to Figure 2

Supplementary Figure S3 is related to Figure 3

Supplementary Figure S4 is related to Figure 4

Supplementary Figure S5 is related to Figure 4

Supplementary Figure S6 is related to Figure 5

Supplementary Figure S7 is the Graphical Abstract

Resources Table

Supplemental datasheet-Table S1 caption, related to Figure 3

Supplemental datasheet-Table S2 caption, related to Figure 4

Supplemental datasheet-Table S3 caption, related to Figure 4

## Supplementary Figure S1


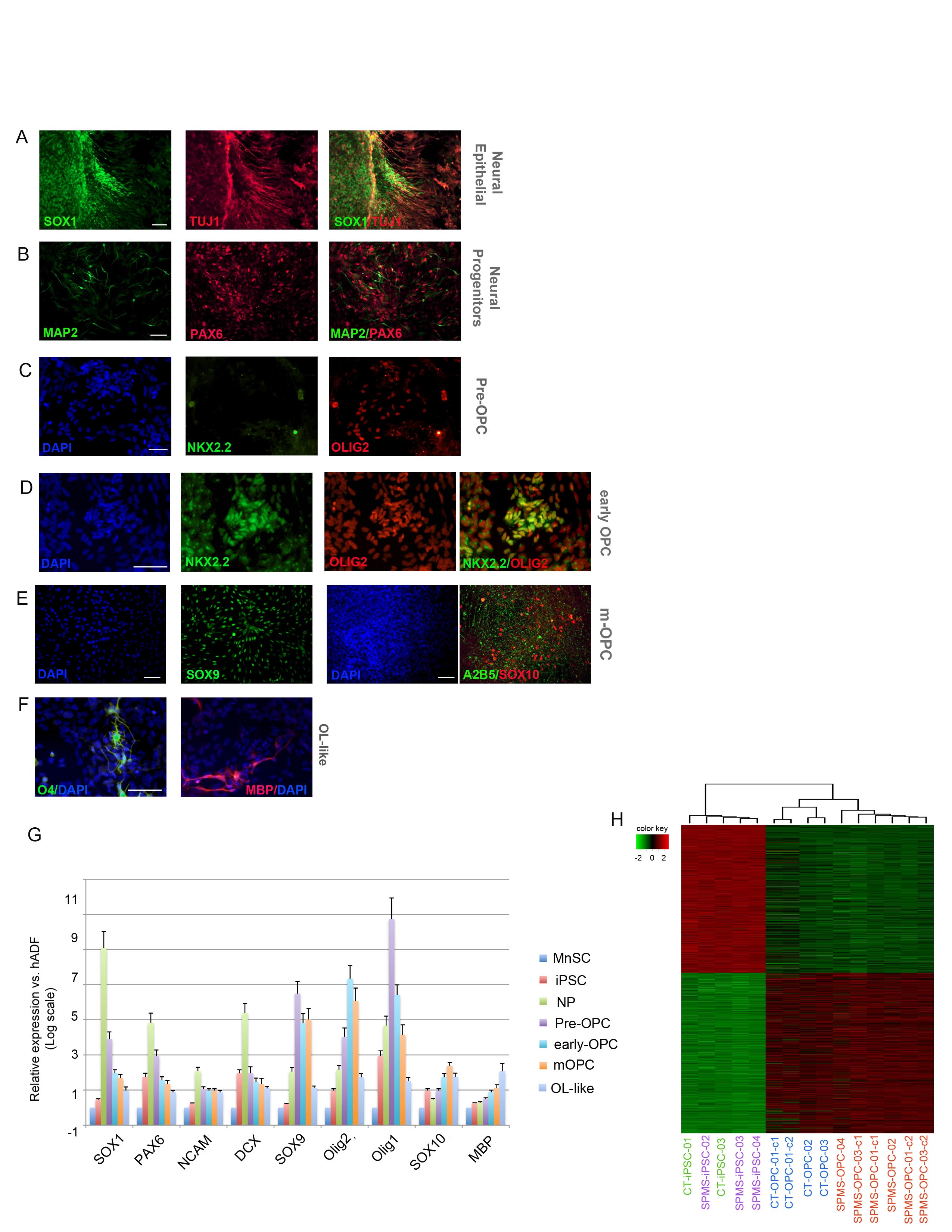


Figure 1. Efficient differentiation of CT-iPS cell lines into oligodendrocyte progenitor-like cell fate. Related to Figure 2. (A-E) Representative immunofluorescence images of CT-iPSCs at each of the differentiation stages underlined: A. CT-iPSC-derived NE cells at this stage expressing the neuroepithelial markers PAX6 and TUJ1; (B). When NE cells are subjected to neural progenitor cells media containing bFGF and EGF they express PAX6 and MAP2 markers; (C). When NE cells are cultured in the absence of bFGF and in presence of RA and SHH agonist purmorphamine they express pre-oligo progenitor (pre-OPC) markers Olig2 but not NKX2.2; (D). After further culture with bFGF and purmorphamine without RA, proliferating early OPC-like cells show Olig2, NKX2.2, SOX9 positive labeling. (E) Culture of early OPC-like cells in glial induction media (GIM) give rise to mature OPC (mOPC) with SOX9, A2B5 and SOX10 positive labeling. (F) After growth factor withdrawal they give rise to oligodendrocyte-like (OL) cells expressing O2 and MBP markers (Scale bar = 50 μm). (G) qRT-PCR for genes characteristic of oligodendroglial fate specification was performed as indicated on mRNA collected from CT-iPS derived cells at indicated stages. Values indicate average relative expression of the specific gene normalized to GAPDH/Actin relative to MnSC expression which was arbitrarily assigned a value of 0, in a logarithmic scale. Data correspond to the average of 3 independent experiments (3 iPSC-CT clones from 3 donors) done in triplicate. (H) Correlation heatmap showing the clustering of iPSC, SPMS- and CT-derived early OPC-like lines (SPMS-OPCMS and CT-OPC) using array-based RNA expression data. Euclidean distance and complete agglomeration method were used to compute the heatmap’s dendrogram. Correlation was computed with Pearson’s method. Related to Figure 2.


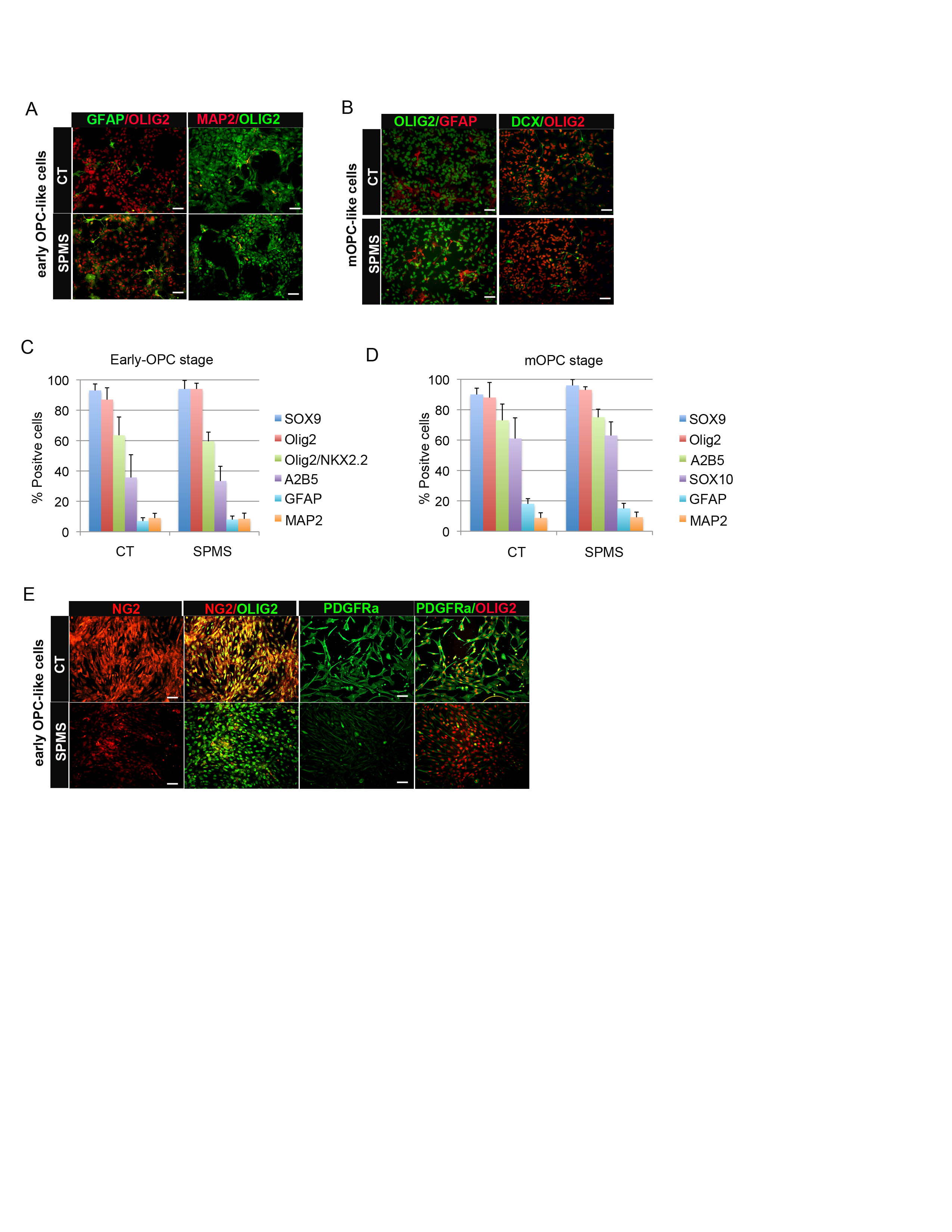


Figure S2. Analysis of cell markers in early OPC-like and mOPC populations at the time of transcriptomic/secretome analysis. (A) Representative immunofluorescence images of CT and SPMS cell types at early OPC and (B) mOPC differentiation stage (as described in supplementary Figure S1) (Scale bar= 50 μm). (C,D) Corresponding image quantification of positive cells for each selected marker shown in figure 2 and supplementary figure S2 A,B done with 3 independent biological replicates.(E) Validation of NG2 and PDGFRα gene expression data in cultures by immunocytofluorescence showing downexpression of both proteins in CT and SPMS derived cells (Scale bar = 50 μm). Related to Figure 2.


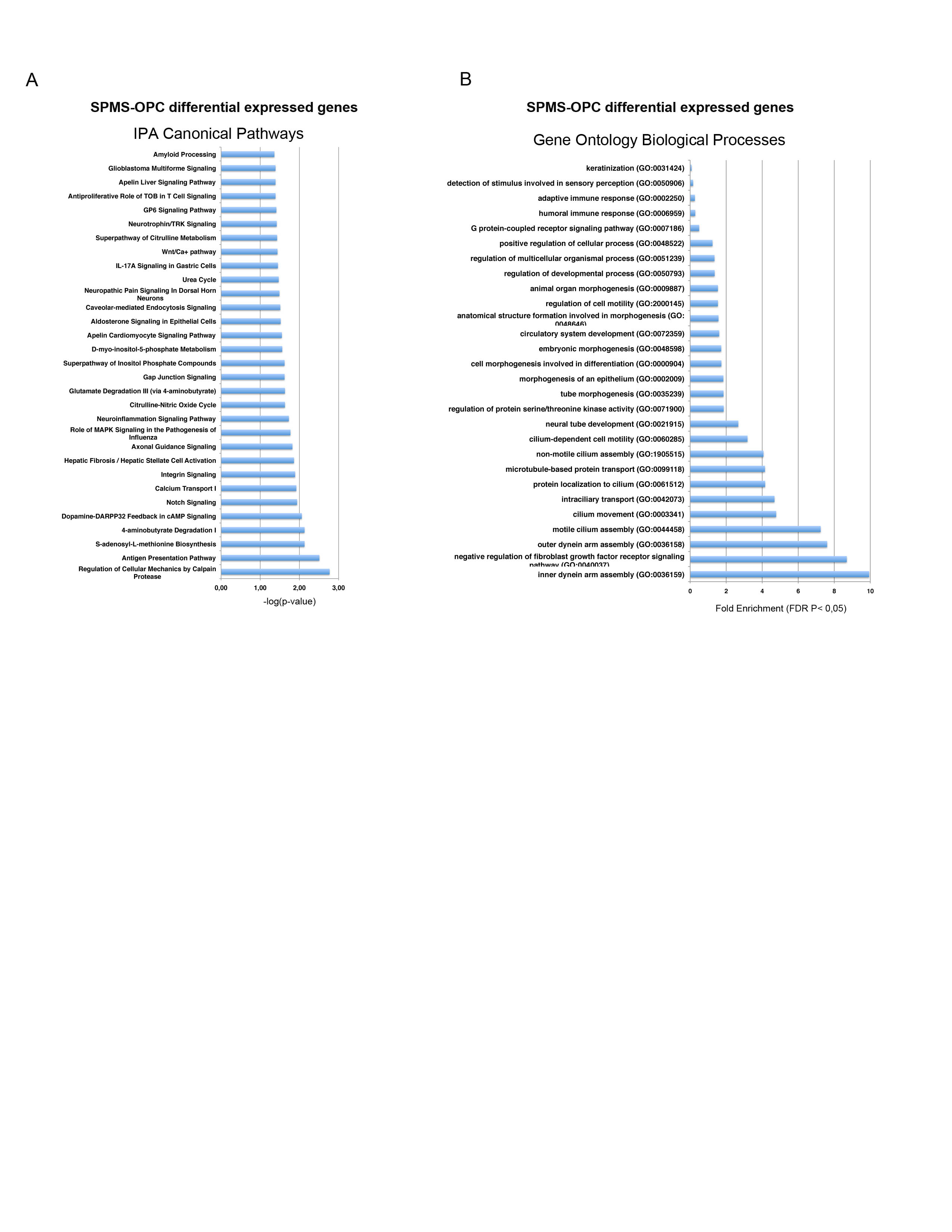


**Figure S3.** Functional Annotation of SPMS-OPC differentially regulated genes. (**A**) Identification of significantly top-represented activated canonical pathways using Ingenuity® Pathway Analysis (IPA®) Software (-log p-value) based on differentially regulated genes between SPMS- and CT-derived early OPC-like cells (as in Figure 3F). (**B**) Significant enriched gene ontologies (GO)- key Biological Processes [1] associated with regulated genes as in Figures S2A (FDR P < 0.05). Related to Figure 3.


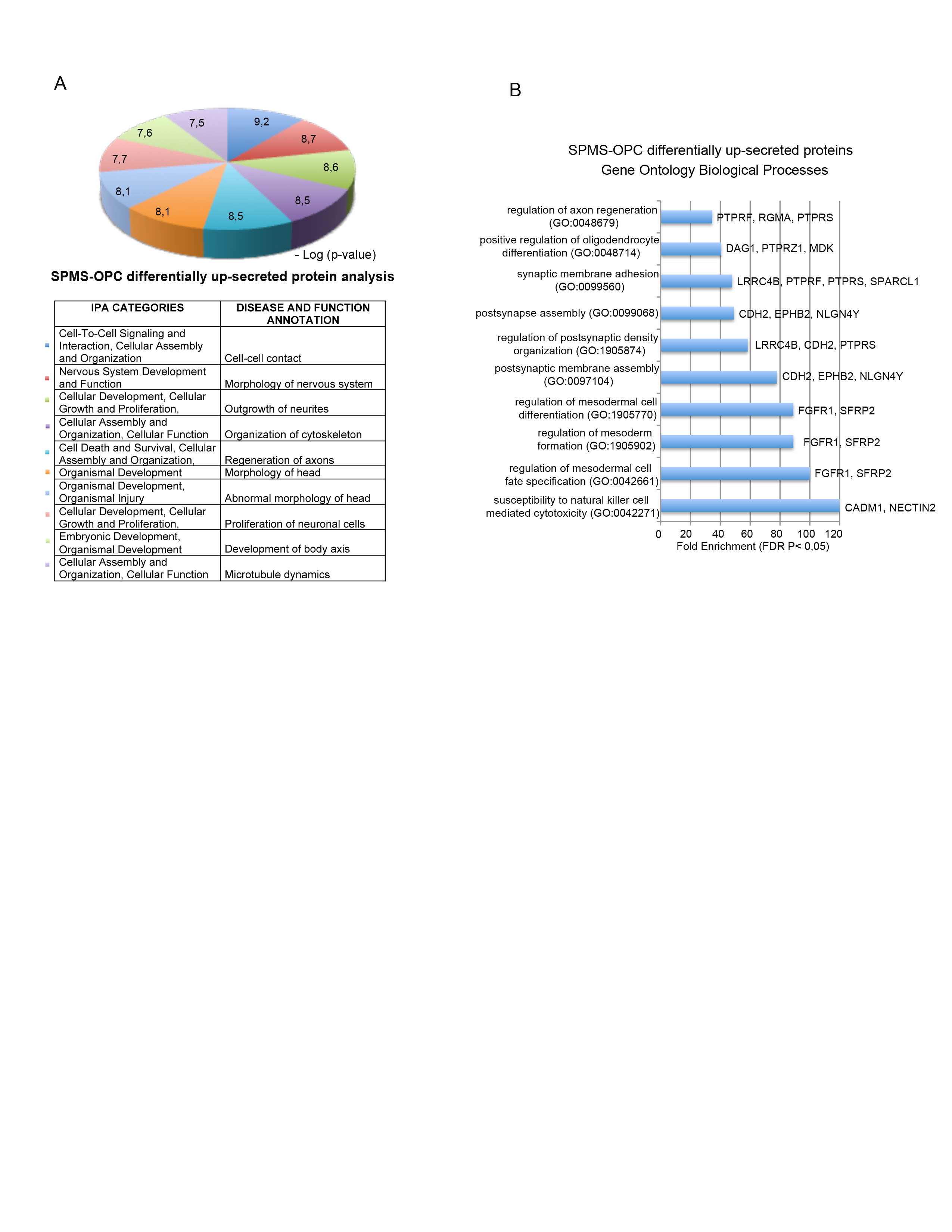


Figure S4. Identification of significantly top-represented disease-and-function categories using Ingenuity® Pathway Analysis (IPA®) (A) or annotated GO Biological Processes (B) Processes [1] based on up-secreted proteins in SPMS-derived early OPC-like cells. Related to Figure 4.


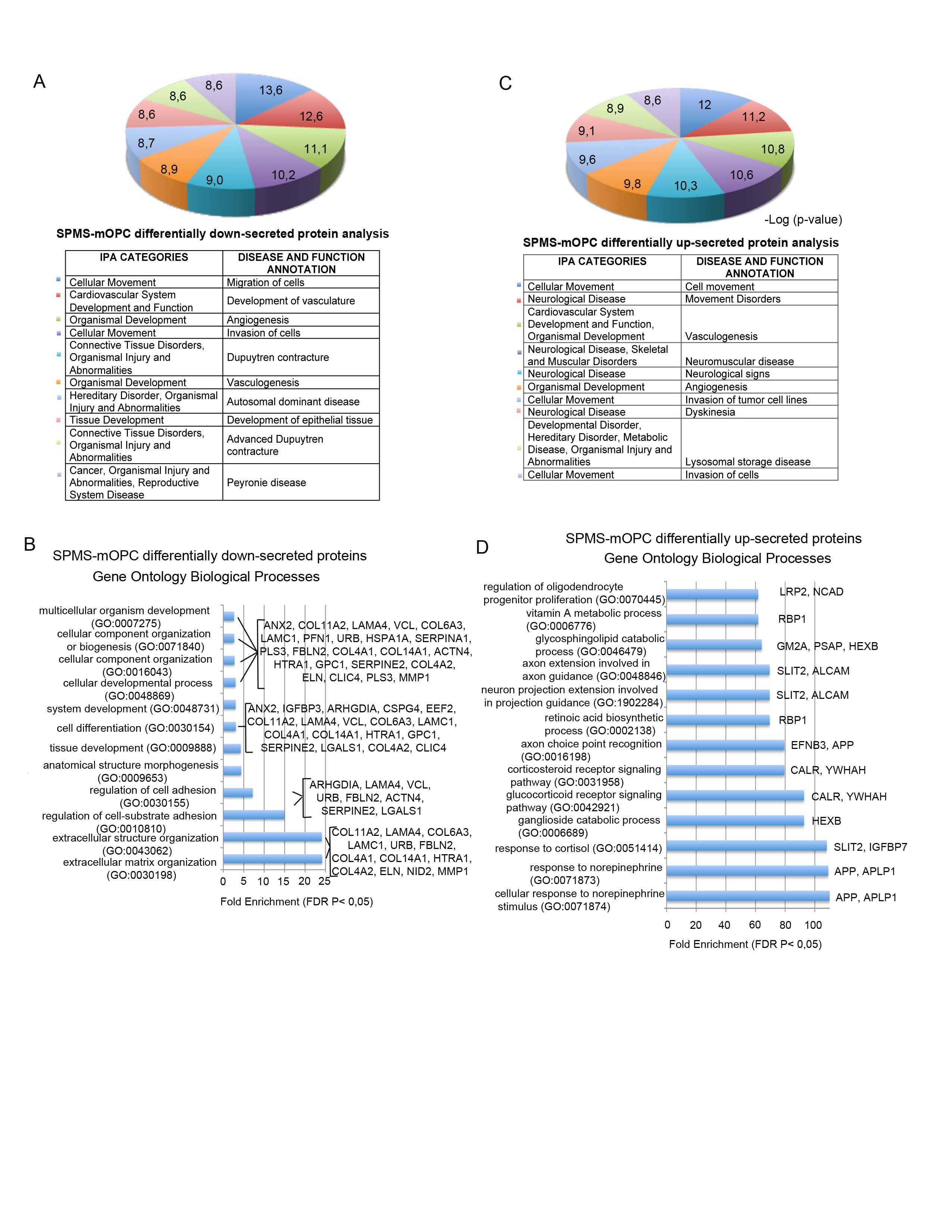


**Figure S5.** Identification of significantly top-represented disease-and-function categories using Ingenuity® Pathway Analysis (IPA®) (**A,C**) or annotated GO Biological Processes (**B,D**) based on down-secreted (**A,B**) or up-secreted (**C,D**) proteins in SPMS-derived mOPC cells. Related to Figure 4.

**Supplementary Figure S6**


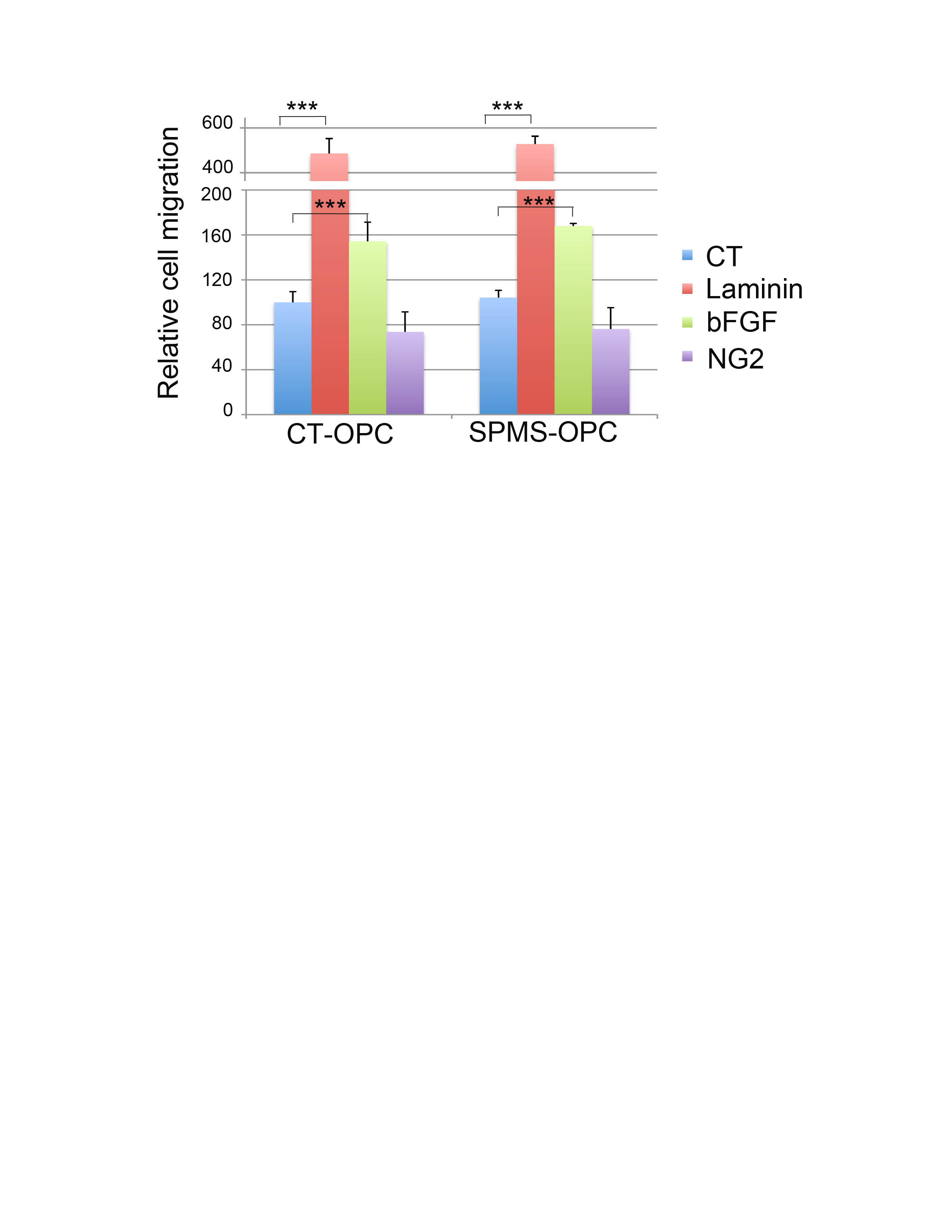


Supplementary Figure S6. OPC-like in vitro cell migration after laminin, bFGF and NG2 stimulation performed in basal media. SPMS- and CT-derived early OPC-like cells were plated on upper wells of transwell chambers and fasting media was added to lower well. 24 hours later, migrated cells on the lower membrane surface were fixed, crystal violet stained and quantified by optical density (OD 540) values of stained cell extracts. Graph shows quantification of cell migration (OD 540) under CT or SPMS-derived CM with indicated stimulating factors (laminin, bFGF or NG2) relative to the CT-OPC-like sample with plain fasting media. Error bars represent means ± SEM (n = 3 independent experiments with early OPC-like cell lines from 4 different SPMS and 3 CT donors) ***P ≤ 0.005 by unpaired Student’s t test (related to Figure 5).

##
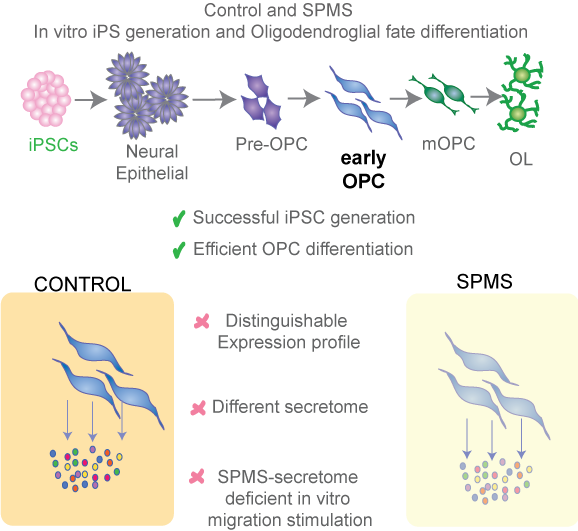


Figure S7. Graphical abstract of main results of the manuscript.

Supplemental datasheet-Table S1: Differential gene expression analysis of SPMS- and CT-derived proliferating early OPC-like cells. Related to Figure 3. Table contains sheets with significant differently regulated genes between both populations. Related to Figure 3.

Supplemental datasheet-Table S2: List of proteins differentially represented in conditional media from SPMS- and CT-derived proliferating early OPC-like cells. Related to Figure 4

Supplemental datasheet-Table S3: List of proteins differentially represented in conditional media from SPMS- and CT-derived mOPC cells. Related to Figure 4.

**Resources Table:**

| **REAGENT or RESOURCE** | | **SOURCE** | | **IDENTIFIER** |  |  |
| --- | --- | --- | --- | --- | --- | --- |
| **Antibodies** | | | | |  |  |
| goat anti-OCT4 | | Santa Cruz Biotechnology | | Cat#sc-8628 |  |  |
| rabbit anti-NANOG | | Santa Cruz Biotechnology | | Cat#sc-33760 |  |  |
| rabbit anti LIN-28 | | Santa Cruz Biotechnology | | Cat#sc-67266 |  |  |
| rabbit anti-SOX2 | | Abcam | | Cat#AB5603 |  |  |
| mouse anti-TRA-1-60 | | Chemicon/Millipore | | Cat#MAB4360 |  |  |
| mouse anti-SSEA4 | | Developmental Studies Hybridoma bank (Iowa) | | Cat#MC-813-70 |  |  |
| goat anti SOX1 | | R&D Systems | | Cat#AF3369 |  |  |
| mouse anti Tuj1 | | Covance | | Cat#MMS-435P |  |  |
| rabbit anti PAX-6 | | StemGent Biolegend | | Cat#901301 |  |  |
| mouse anti-MAP2 (AP20 | | Santa Cruz Biotechnology | | Cat#sc-32791 |  |  |
| rabbit anti Olig2 | | Chemicon/Millipore | | Cat#AB9610 |  |  |
| mouse anti-NKX2.2 | | Developmental Studies Hybridoma bank (Iowa) | | Cat#74.5A5 |  |  |
| goat anti-SOX9 | | R&D Systems | | Cat#AF3075 |  |  |
| mouse anti O4 (clone 81) | | Chemicon/Millipore | | Cat#MAB345 |  |  |
| rabbit anti-Myelin Basic Protein (MPB) [EPR21188] | | Abcam | | Cat#ab218011 |  |  |
| mouse anti A2B5 | | Chemicon/Millipore | | Cat#MAB312R |  |  |
| rabbit anti-SOX10 [SP267] | | Abcam | | Cat#ab227680 |  |  |
| rabbit anti-DCX | | Abcam | | Cat#AB18723 |  |  |
| rabbit anti-GFAP | | Agilent-DAKO | | Cat#M076101-2 |  |  |
| Alexa Fluor® 488 donkey anti mouse IgG (H+L) | | Life Technologies | | Cat#A21202 |  |  |
| Alexa Fluor® 488 donkey anti rabbit IgG (H+L) | | Life Technologies | | Cat#A21206 |  |  |
| Alexa Fluor 555 donkey anti-rabbit IgG (H+L) | | Life Technologies | | Cat#A31572 |  |  |
| Alexa Fluor 555 donkey anti-mouse IgG (H+L) | | Life Technologies | | Cat#A31570 |  |  |
| Alexa Fluor® 488 donkey anti-goat IgG (H+L) | | Life Technologies | | Cat#A11055 |  |  |
| Peroxidase AffiniPure Goat Anti-Rabbit IgG (H+L) | | Jackson InmunoResearch | | Cat#111-035-144 |  |  |
| Peroxidase AffiniPure Goat Anti-Mouse IgG (H+L) | | Jackson InmunoResearch | | Cat#115-035-003 |  |  |
|  | |  | |  |  |  |
|  | |  | |  |  |  |
| **Vectors and plasmids** | | | | |  |  |
|  | |  | |  |  |  |
| pMXs-GFP | | Cell Biolabs, INC | | Cat#RTV-053 |  |  |
| pMXs-hOCT4 | | [2] | | Addgene-Cat#17217 |  |  |
| pMXs-hSOX2 | | [2] | | AddgeneCat#17218 |  |  |
| pMXs-hKLF4 | | [2] | | AddgeneCat#17219 |  |  |
| pMXs-hcMYC | | [2] | | Addgene Cat#17220 |  |  |
| Gag/Pol | | [3] | | Addgene Cat#14887 |  |  |
| pCMV-VSV-G | | [4] | | Addgene Cat#8454) |  |  |
|  | |  | |  |  |  |
| **Chemicals, Peptides, and Recombinant Proteins** | | | | |  |  |
|  | |  | |  |  |  |
| KO_DMEM | | Thermo Fisher Scientific | | Cat#10829018 |  |  |
| DMEM/F12 | | Thermo Fisher Scientific | | Cat#11320-082 |  |  |
| Neurobasal Medium | | Thermo Fisher Scientific | | Cat#21103049 |  |  |
| MEM Non-Essential Amino Acids Solution (100X) | | Thermo Fisher Scientific | | Cat#11140068 |  |  |
| L-Glutamine | | Thermo Fisher Scientific | | Cat# 21051024 |  |  |
| Penicillin-Streptomycin | | Thermo Fisher Scientific | | Cat# 15070063 |  |  |
| 2-mercaptoethanol | | Thermo Fisher Scientific | | Cat# 21985023 |  |  |
| Fetal Bovine Serum, Regular (Heat Inactivated) | | Corning | | Cat# 35-011-CV |  |  |
| KO-Serum Replacement | | Thermo Fisher Scientific | | Cat# 10828028 |  |  |
| 2-mercaptoethanol | | Thermo Fisher Scientific | | Cat# 21985023 |  |  |
| Recombinant human basic FGF-premium grade | | MACS-Miltenyi Biotec | | Cat#130-093-843 |  |  |
| Y-27632 | | Tocris Biosciences | | Cat# 1254 |  |  |
| Mitomycin C from Streptomyces caespitosus | | Sigma-Aldrich | | M4287 |  |  |
| Matrigel hESC-qualified matrix | | Corning | | Cat# 354277 |  |  |
| N-2 Supplement (100X) | | Thermo Fisher Scientific | | Cat# 17502048 |  |  |
| B-27 Supplement (50X), minus vitamin A | | Thermo Fisher Scientific | | Cat# 12587010 |  |  |
| Laminin | | Thermo Fisher Scientific | | Cat# 23017015 |  |  |
| poly-L-ornithine | | Sigma-Aldrich | | Cat# # P4957 |  |  |
| Gelatin Solution | | Sigma-Aldrich | | Cat#G1393 |  |  |
| Purmorphamine | | Sigma-Aldrich | | Cat#540220 |  |  |
| Epidermal growth factor (EGF) | | Peprotech | | Cat#AF-100-15 |  |  |
| GlutaMAX™ Supplement | | Gibco-ThermoFisher | | Cat# 35050061 |  |  |
| Accutase solution | | Sigma-Aldrich | | Cat# A6964-100ML |  |  |
| Polybrene | | Sigma-Aldrich | | Cat#H9268-10G |  |  |
| Venor GeM Classic | | Minerva Biolabs | | Cat#11-1050 |  |  |
| cOmplete™ Protease Inhibitor Cocktail | | MERCK | | Cat#11697498001 |  |  |
| Igepal (Nodidet P40 subsitute) | | MERCK | | Cat#11332473001 |  |  |
| Crystal violet solution 1% aqueous solution | | Sigma-Aldrich | | Cat#V5265-250ML |  |  |
| DAPI | | Sigma-Aldrich | | Cat#D9542 |  |  |
| BSA | | Sigma-Aldrich | | Cat#:A4503 |  |  |
| Paraformaldehyde | | Electron Microscopy Sciences | | Cat#:15710 |  |  |
| DPBS | | Corning | | Cat#:21-031-CM |  |  |
| trichloroacetic acid | | Sigma-Aldrich | | Cat#T0699-100ML |  |  |
| Sodium orthovanadate | | Sigma-Aldrich | | Cat#S6508-10G |  |  |
| Sodium deoxycholate | | Sigma-Aldrich | | Cat#D6750 |  |  |
| Polyethylenimine | | Polysciences Inc., | | Cat# 23966 |  |  |
| Donkey Serum | | Equitech-Bio | | Cat#:SD30-0500 |  |  |
| Triton X-100 | | Fisher Scientific | | Cat#:BP151-500 |  |  |
| Normal Donkey Serum | | Sigma Aldrich | | Cat#D9663 |  |  |
| Retinoic Acid | | Sigma Aldrich | | Cat#R2625 |  |  |
| NG2 recombinant protein | | R&D (Research And Diagnostic) Systems | | Cat#2585-PG-050 |  |  |
| IGF-1 human recombinant protein | | Peprotech | | Cat#100-11 |  |  |
| PDGF AA human recombinant protein | | Peprotech | | Cat# 100-13A |  |  |
| NT3 human recombinant protein | | Sigma Aldrich | | Cat#T6397 |  |  |
| biotin | | Sigma Aldrich | | Cat#B4501 |  |  |
| dibutyryl-cAMP | | Sigma Aldrich | | Cat#D0260 |  |  |
| **Critical Commercial Assays** | | | | |  |  |
|  | |  | |  |  |  |
| SYBR Green PCR Master Mix | | Applied Biosystems | | Cat#4309155 |  |  |
| QIAGEN Gentra Puregene Cell Kit | | QIAGEN | | Cat#: 158388 |  |  |
| RNeazy Mini Plus Kit QIAGEN | | QIAGEN | | Cat# 74136 |  |  |
| Qubit dsDNA BR Assay Kits | | Life Technologies | | Cat#Q32853 |  |  |
| Human Clarion-S Microarrays Affymetrix | | Applied Biosystems | | Cat#902927 |  |  |
| Illumina Human HT-12 v4.0 Expression BeadChip | | Illumina | | Cat#Human HT-12 v4.0 |  |  |
| MTT assay | | Sigma Aldrich | | Cat#M2128 |  |  |
| 12 well-transwell chamber with 8 μm pore-size | | Corning | | Cat#3403 |  |  |
| Merck Millipore Amicon™ Ultra Centrifugal Filter Units 3 MW | | Fisher Scientific | | Cat#10403892 |  |  |
| **Deposited Data** | | | | |  |  |
| RNA-array of MnSCs, iPSCs and early OPC cells | | This paper | | GSE151306 |  |  |
|  | |  | |  |  |  |
| **Experimental Models: Cell Lines** | | | | |  |  |
| Human: HEK293T/17 cells ATCC Cat# CRL | | 11268; RRID: CVCL_1926 | | Human: HEK293T/17 cells ATCC Cat# CRL |  |  |
| Human: Passage 33 and 67 H9 human ES | | WiCell Research Institute | | Cat#WA09 |  |  |
| Human female control donor 01: adult menstrual derived stromal cells_control 01 | | This study | | CT-MnSC-01 |  |  |
| Human female control donor 01: adult menstrual derived stromal cells_control 02 | | This study | | CT-MnSC-02 |  |  |
| Human female control donor 01: adult menstrual derived stromal cells_control 03 | | This study | | CT-MnSC-03 |  |  |
| Human female SPMS donor 01: adult menstrual derived stromal cells_SPMS 01 | | This study | | SPMS-MnSC-01 |  |  |
| Human female SPMS donor 01: adult menstrual derived stromal cells_SPMS 02 | | This study | | SPMS-MnSC-02 |  |  |
| Human female SPMS donor 01: adult menstrual derived stromal cells_SPMS 03 | | This study | | SPMS-MnSC-03 |  |  |
| Human female SPMS donor 01: adult menstrual derived stromal cells_SPMS 04 | | This study | | SPMS-MnSC-04 |  |  |
| CT-iPS cell lines (OSK reprogramming) from somatic samples CT-MnSC-01 to -03 | | This study | | CT-iPSC-01 to -03 |  |  |
| SPMS-iPS cell lines (OSK reprogramming) from somatic samples CT-MnSC-01 to -04 | | This study | | SPMS-iPSC-01 to -04 |  |  |
| Early OPC cell lines derived from each CT-iPS cell line 01 to 03 | | This study | | CT-OPC-01 to -04 |  |  |
| Early OPC cell lines derived from each SPMS-iPS cell line 02 and 04 | | This study | | SPMS-OPC-02 and SPMS-OPC-04 |  |  |
| Early OPC cell lines derived from SPMS-iPS cell line 01 | | This study | | SPMS-OPC-01-c1 and SPMS-OPC-01-c2 |  |  |
| Early OPC cell lines derived from SPMS-iPS cell line 03 | | This study | | SPMS-OPC-03-c1 and SPMS-OPC-03-c2 |  |  |
|  | |  | |  |  |  |
|  | |  | |  |  |  |
| **Experimental Models: Organisms/Strains** | | | | |  |  |
| NOD SCID Mouse Congenic Immunodeficient | | Charles River | | NOD.CB17-Prkdcscid/NCrCrl |  |  |
|  | |  | |  |  |  |
| **Oligonucleotides** | | | | |  |  |
|  |  | |  | |  |  |
|  | Forward | | Reverse | |  |  |
| RT-NANOG | TACCTCAGCCTCCAGCAGAT | | TCTGGAACCAGGTCTTCACC | |  |  |
| RT POU5F1 endog | CCTCACTTCACTGCACTGTA | | CAGGTTTTCTTTCCCTAGCT | |  |  |
| RT POU5F1 transgene | CCCCAGGGCCCCATTTTGGTACC | | CTTCCCTCCAACCAGTTGCCCCAAAC | |  |  |
| RT POU5F1 | GGTTCTATTTGGGAAGGTAT | | CATGTTCTTGAAGCTAAGC | |  |  |
| RT LIN28 |  | |  | |  |  |
| RT-DNMT3B | ATAAGTCGAAGGTGCGTCGT | | GGCAACATCTGAAGCCATTT | |  |  |
| RT-hTERT | TGTGCACCAACATCTACAAG | | GCGTTCTTGGCTTTCAGGAT | |  |  |
| RT-hGDF3 | AAATGTTTGTGTTGCGGTCA | | TCTGGCACAGGTGTCTTCAG | |  |  |
| RT-SOX2 | CCCAGCAGACTTCACATGT | | CCTCCCATTTCCCTCGTTTT | |  |  |
| RT-KLF4 | GATGAACTGACCAGGCACTA | | GTGGGTCATATCCACTGTCT | |  |  |
| RT-ZFP42 | CCCACAGTCCATCCTTACAGAGTT | | GGG ACT TTG CCC CCA AAC | |  |  |
| RT-ACTIN | TGAAGTGTGACGTGGACATC | | GGAGGAGCAATGATCTTGAT | |  |  |
| RT-GAPDH | ATGGAAATCCCATCACCATCTT | | CGG CCC ACT TGA TTT TGG | |  |  |
| RT-TBP | CGGCTGTTTAACTTCGCTTC | | CACACGCCAAGAAACAGTGA | |  |  |
| RT-SOX1 | CACAACTCGGAGATCAGCAA | | GGTACTTGTAATCCGGGTGC | |  |  |
| RT-PAX6 | CGGAGTGAATCAGCTCGGTG | | CCGCTTATACTGGGCTATTTTGC | |  |  |
| RT-DCX | AACTCTACACTCTGGATG | | CTCGGCATTCATTTTCAT | |  |  |
| RT-SOX9 | GACCAGTACCCGCACTTG | | GCTCTCGTTCAGAAGTCTC | |  |  |
| RT-Olig2 | GAAACTACCCCACCGACTCA | | ACCCAAACTGTTTCCACAGC | |  |  |
| RT-Olig1 | CCCCAAAAGTAGCGTAACCA | | GCGGTTGGTTTTCGTTTTTA | |  |  |
| RT-SOX10 | ATCCAGGCCCACTACAAGAG | | GAAGTCGATGTGAGGCTTCC | |  |  |
| RT-BMP | AAGGCCAGAGACCAGGATTT | | TAGGTAACAGGGGCAAGTGG | |  |  |
| RT-PDGFRα | CCTTGGTGGCACCCCTTAC | | TCCGGTACCCACTCTTGATCTT | |  |  |
| RT-RUNX1 | CCCTAGGGGATGTTCCAGAT | | TGAAGCTTTTCCCTCTTCCA | |  |  |
| RT- BRACHYURY | ACCACCGCTGGAAATATGTGAACG | | AACTCTCACGATGTGAATCCGAGG | |  |  |
| RT-GATA4 | CTCTACATGAAGCTCCAC | | CTGCTGGTGTCTTAGATT | |  |  |
| RT-AFP | AGCTTGGTGGTGGATGAAAC | | CCCTCTTCAGCAAAGCAGAC | |  |  |
| RT-NESTIN | CAGCGTTGGAACAGAGGTTGG | | TGGCACAGGTGTCTCAAGGGTAG | |  |  |
| RT-NCAM | ATGGAAACTCTATTAAAGTGAACCTG | | TAGACCTCATACTCAGCATTCCAGT | |  |  |
|  |  | |  | |  |  |
| Software and Algorithms | |  | |  |  |  |
|  | | | | |  |  |
| Affymetrix ® GeneChip® Command Console® 2.0 software | | Affymetrix (Thermo Fisher) | | N/A |  |  |
| R package limma v3.32.3 | | [5] | | https://bioconductor.org/packages/release/bioc/html/limma.html |  |  |
| R package RnBeads | | [6] | | https://bioconductor.org/packages/release/bioc/html/RnBeads.html |  |  |
| Ggplot2 R package | | [7] | | https://www.bioconductor.org/packages/devel/bioc/vignettes/sights/inst/doc/sights.html |  |  |
| GAMP R package | | [8]. | | http://bioconductor.org/packages/release/bioc/html/methylPipe.html |  |  |
|  | |  | |  |  |  |
|  | |  | |  |  |  |

References

1. The Gene Ontology, C. The Gene Ontology Resource: 20 years and still GOing strong. *Nucleic Acids Res.* **2019**, *47*, D330–D338, doi:10.1093/nar/gky1055.

2. Takahashi, K.; Tanabe, K.; Ohnuki, M.; Narita, M.; Ichisaka, T.; Tomoda, K.; Yamanaka, S. Induction of pluripotent stem cells from adult human fibroblasts by defined factors. *Cell* **2007**, *131*, 861–872, doi: 10.1016/j.cell.2007.11.019.

3. Reya, T.; Duncan, A.W.; Ailles, L.; Domen, J.; Scherer, D.C.; Willert, K.; Hintz, L.; Nusse, R.; Weissman, I.L. A role for Wnt signalling in self-renewal of haematopoietic stem cells. *Nature* **2003**, *423*, 409–414, doi:10.1038/nature01593.

4. Stewart, S.A.; Dykxhoorn, D.M.; Palliser, D.; Mizuno, H.; Yu, E.Y.; An, D.S.; Sabatini, D.M.; Chen, I.S.; Hahn, W.C.; Sharp, P.A., et al. Lentivirus-delivered stable gene silencing by RNAi in primary cells. *RNA* **2003**, *9*, 493–501, doi:10.1261/rna.2192803.

5. Ritchie, M.E.; Phipson, B.; Wu, D.; Hu, Y.; Law, C.W.; Shi, W.; Smyth, G.K. limma powers differential expression analyses for RNA-sequencing and microarray studies. *Nucleic Acids Res.* **2015**, *43*, e47, doi:10.1093/nar/gkv007.

6. Assenov, Y.; Muller, F.; Lutsik, P.; Walter, J.; Lengauer, T.; Bock, C. Comprehensive analysis of DNA methylation data with RnBeads. *Nat. Methods* **2014**, *11*, 1138–1140, doi:10.1038/nmeth.3115.

7. Wickham, H. ggplot2: Elegant Graphics for Data Analysis, Use R! *Springer-Verlag, New York.* **2009**.

8. Zhao, N.; Bell, D.A.; Maity, A.; Staicu, A.M.; Joubert, B.R.; London, S.J.; Wu, M.C. Global analysis of methylation profiles from high resolution CpG data. *Genet Epidemiol* **2015**, *39*, 53–64, doi:10.1002/gepi.21874.
